# Interactions of Biodegradable Ionic Liquids with a Model Naphthenic Acid

**DOI:** 10.1038/s41598-017-18587-1

**Published:** 2018-01-09

**Authors:** Chongchong Wu, Alex De Visscher, Ian Donald Gates

**Affiliations:** 1 Department of Chemical and Petroleum Engineering, University of Calgary, Alberta, Canada; 2 Department of Chemical and Materials Engineering, Concordia University, Montreal, Quebec, Canada

## Abstract

Density functional theory models are used to examine five biodegradable ionic liquids (ILs) each one consisting of a substitutional group (-OH, -NH_2,_ -COOH, -COOCH_3_, and -OCH_3_) incorporated into the cation of 1-butyl-3-methylimidazolium tetrafluoroborate ([BMIM][BF_4_]). The results reveal that hydrogen atoms in -NH_2,_ -COOH, and -COOCH_3_ form intramolecular hydrogen bonds with fluorine atoms in [BF_4_]^−^, whereas hydrogen atoms in -OH and -OCH_3_ do not form hydrogen bonds with [BF_4_]^−^. Further analysis of electron density at bond critical points and noncovalent interactions suggest that [BMIM][BF_4_] with -COOH has stronger intramolecular hydrogen bonds than other ILs. The extraction mechanism for a model naphthenic acid is hydrogen bonding, with F···H being the strongest hydrogen bond and O···H ranking second. More intermolecular hydrogen bonds occur when model naphthenic acid is adsorbed by [BMIM][BF_4_] with -COOH and -COOCH_3_. The interaction energy between model naphthenic acid and ILs with -COOH and -COOCH_3_ is higher than that with -OH, -NH_2_, and -OCH_3_.

## Introduction

Naphthenic acids (NAs) are complex mixtures of carboxylic acids with cyclic structures and aliphatic groups^1,2^. In addition to increasing the acidity of crude oils, the presence of NAs also cause other serious problems such as poisoning catalysis, forming coke, and creating corrosion to equipment and pipelines^3,4^. Ionic liquids (ILs), recognized as “green solvents” and “solvents of the future”, provide us an alternative and promising approach to extract undesired products from fossil fuels, considering their outstanding properties such as non-volatility, low flammability, and reusability^5–7^. It was reported that ILs are efficient at removing NAs from crude oil^8–10^.

Having excellent physicochemical properties and being easily synthesized, imidazolium-based liquids are the most extensively studied ILs^11^. However, the properties (thermal stability and non-volatility) that make ILs attractive result in low biodegradation of ILs^12,13^. Due to their high solubility, high stability, and low biodegradability, imidazolium-based ILs are persistent pollutants that cause serious contamination after being released to aqueous media^14,15^. ILs are also reported to be toxic to a broad variety of organisms^16^. The most effective technique to remove organic pollutants from water is biodegradation^17^, hence, a significant challenge associated with the design and application of ILs is to increase the biodegradability of ILs^18^.

To improve the ultimate biodegradation of ILs and minimize the adverse environmental influences, researchers have paid more attention to change the framework of organic cations and anions of ILs. It has been demonstrated that incorporating an ester in the side chain significantly enhanced imidazolium-based ILs biodegradation^19^. Furthermore, oxygenated and hydroxylated imidazolium-based ILs have a better biodegradability^20^. Amino groups are also reported to be able to increase the biodegradability of ILs^21^. Although there are numerous studies to investigate approaches to increase the biodegradability of ILs, to the best of our knowledge, few theoretical studies are available to compare intramolecular and intermolecular interaction differences, and extraction mechanism variations of ILs with different biodegradable substitutional groups, especially for the removal of NAs from liquid oil.

**Figure 1:**
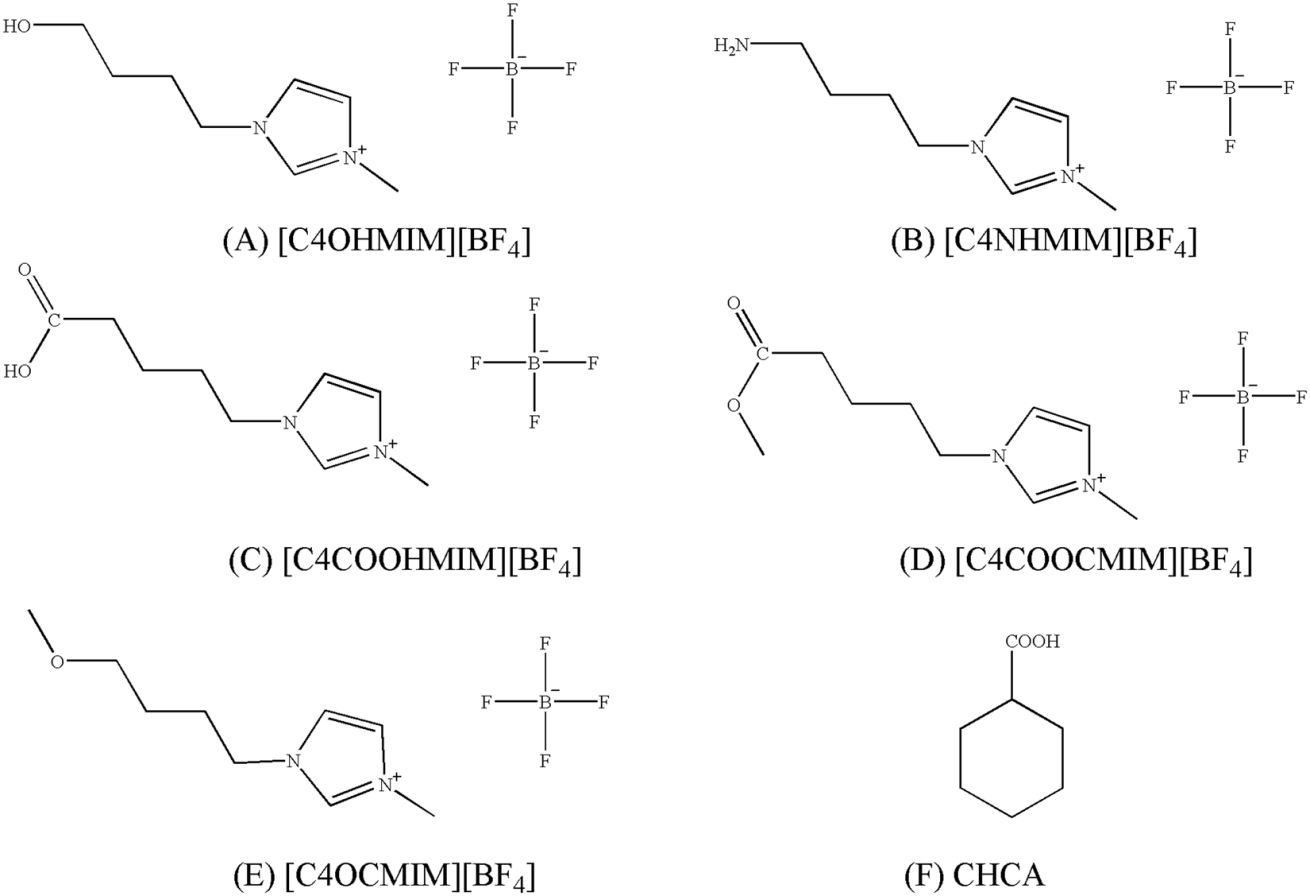
Chemical structures of ILs with biodegradable groups and CHCA.

The objective of the research was to fill the knowledge gap by exploring the influence of biodegradable substitutional groups on ILs intramolecular interactions, examining ILs extraction mechanisms of model NAs, and investigating the nature of the molecular interactions by using density functional theory (DFT) calculation. Five different biodegradable groups, including the hydroxyl group (-OH), amino group (-NH_2_), formate group (-COOH), methyl ester group (-COOCH_3_), and methyl ether group (-OCH_3_) were incorporated to the cation of 1-butyl-3-methylimidazolium tetrafluoroborate ([BMIM][BF_4_]). The structures of the five ILs with these biodegradable groups are displayed in Fig. 1. Cyclohexanecarboxylic acid (CHCA) was selected to be the model NA. The results reported in this study will assist researchers to design ILs with high biodegradability and high extraction efficiency for NAs.

## Results and Discussion

### Optimized Geometries

The most stable geometries of CHCA and ILs with biodegradable groups are shown in Supplementary Fig. S1, with bond lengths listed in Supplementary Table S1. It is illustrated that [BF_4_]^−^ is located above the imidazole ring for all these stable structures of ILs, which is identical with [BMIM][BF_4_], demonstrating that the incorporation of biodegradation groups do not remarkably change the relative position of [BF_4_]^-^ to the cations^22^. The Van Der Waals (VDW) radii for nitrogen, oxygen, fluorine, and hydrogen are reported to be 1.55, 1.52, 1.47, and 1.2 Å, respectively^23^. For [C4OHMIM][BF_4_], the distances of O30···H20, O30···H21, O30···H28, and O30···H29 are 2.53, 2.61, 2.08, and 2.08 Å, respectively (Supplementary Table S1), which are shorter than the sum of the VDW radii for oxygen and hydrogen. Consequently, it is concluded that hydrogen bonds are formed between the oxygen atom in -OH and hydrogen atoms of [C4OHMIM][BF_4_]. In addition, the nitrogen atom in -NH_2_ and oxygen atoms in -COOCH_3_, -COOH, and -OCH_3_ form hydrogen bonds with hydrogen atoms in the ILs as well. Moreover, hydrogen bonding also occurs between fluorine atoms in [BF_4_]^-^ and hydrogen atoms in the cation for all these five types of ILs (Supplementary Fig. S1 and Table S1), which follows the same trends as [BMIM][BF_4_]^22^. Interestingly, different from -OH and -OCH_3_, the distances of F24···H31 (2.53 Å) in [C4NHMIM][BF_4_], F31···H28 (1.99 Å) in [C4COOHMIM][BF_4_], and F32···H31 (2.20 Å) in [C4COOCMIM][BF_4_] are shorter than the VDW radius for fluorine and hydrogen, indicating the existence of intramolecular hydrogen bonds between a fluorine atom of [BF_4_]^−^ and a hydrogen atom of -NH_2,_ -COOH, and -COOCH_3_ for those three type of ILs. On the other hand, the interactions between a fluorine atom in [BF_4_]^−^ and a carbon atom in the imidazole ring corresponds to the presences of lone pair (LP)-π interactions.

**Figure 2:**
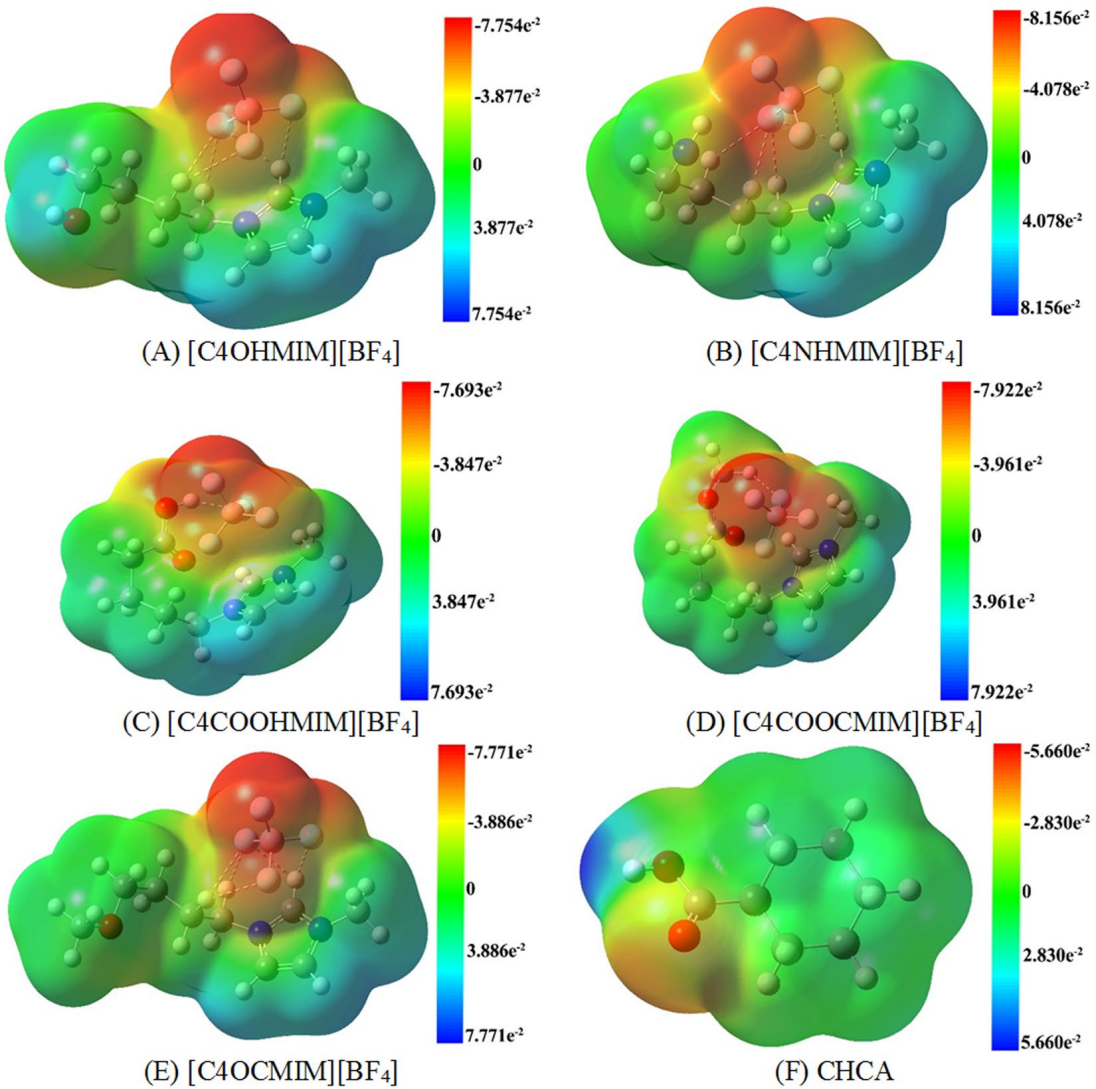
The electrostatic potential (a.u.) of (**A**) [C4OHMIM][BF_4_], (**B**) [C4NHMIM][BF_4_], (**C**) [C4COOHMIM][BF_4_], (**D**) [C4COOCMIM][BF_4_], and (**E**) [C4OCMIM][BF_4_].

To efficiently acquire the most stable interaction structures for ILs-CHCA, the electrostatic potential was analyzed for ILs and CHCA, shown in Fig. 2. It was deduced that the highly positively charged regions for ILs are located around the imidazole ring, whereas [BF_4_]^−^ has a strongly negatively electrostatic potential. The addition of -OH and -OCH_3_ negatively influences the electrostatic potential, and those regions have stronger negative electrostatic potential compared with that for [BMIM][BF_4_]^22^. With regards to CHCA, the most negative and positive electrostatic potentials are located around the oxygen atoms in the carboxylic group, and the hydrogen atom in the carboxylic group, separately.

**Figure 3:**
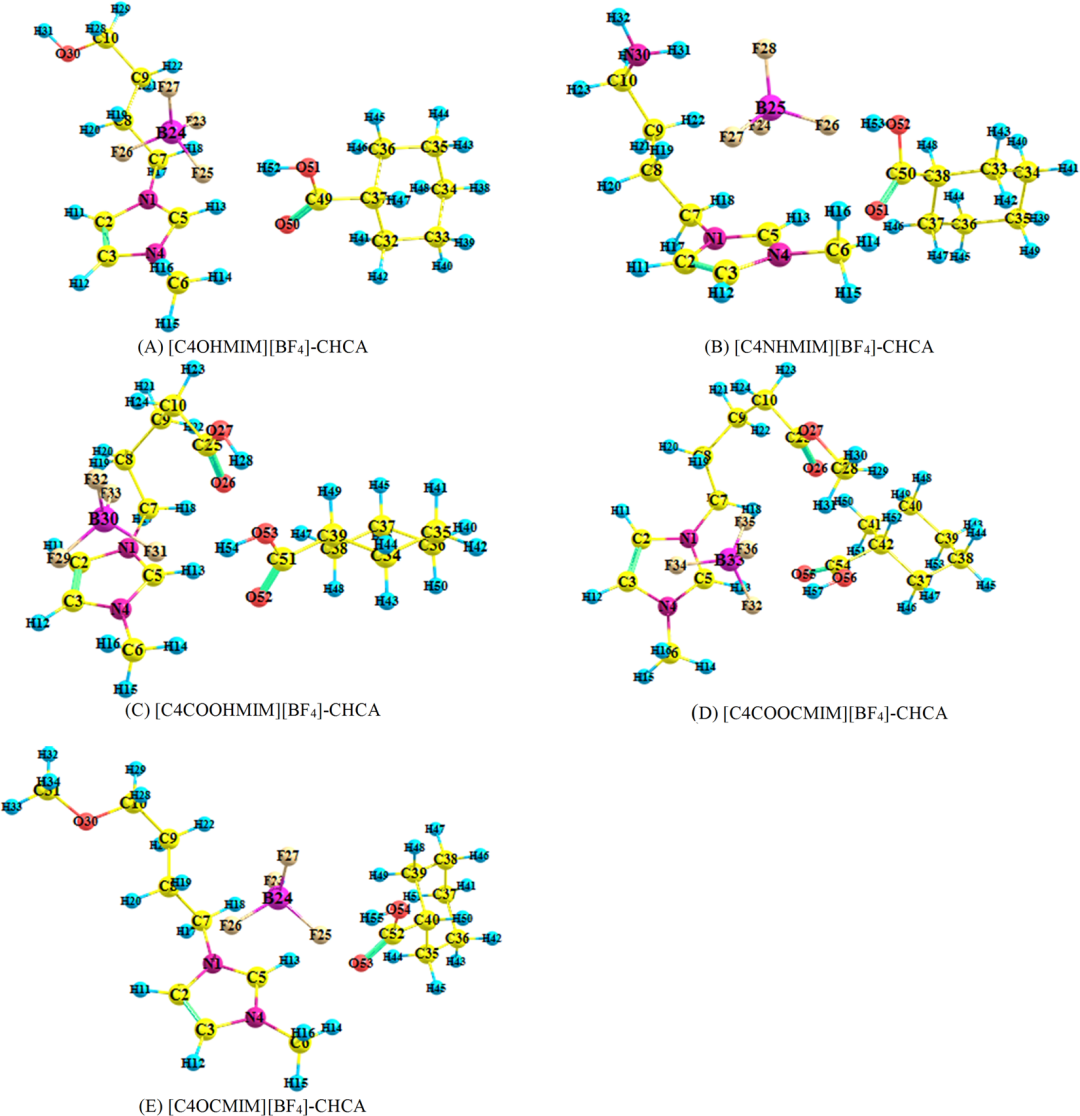
The optimized structures of (**A**) [C4OHMIM][BF_4_]-CHCA, (**B**) [C4NHMIM][BF_4_]-CHCA, (C) [C4COOHMIM][BF_4_]-CHCA, (**D**) [C4COOCMIM][BF_4_]-CHCA, and (**E**) [C4OCMIM][BF_4_]-CHCA.

Based on electrostatic potential analysis, CHCA were placed around different regions of ILs to obtain the most stable interaction structures, as shown in Fig. 3 with Cartesian coordinates in Supplementary Table S2. There are three dominant hydrogen bonds for [C4OHMIM][BF_4_]-CHCA, one is between a fluorine atom of [BF_4_]^−^ and the hydrogen atom in the carboxylic group of CHCA; the other two are between an oxygen atom in the carboxylic group of CHCA and hydrogen atoms of the cation, presented in Fig. 3A and Table 1. With the formation of complexes, the distance of F25···H14 (displayed in Supplementary Fig. S1 and Table S1) is lengthened from 2.61 Å to 2.79 Å, as displayed in Fig. 3A and Table 1, longer than the sum of VDW radii for the fluorine and hydrogen atoms, indicating that F25 no longer forms a hydrogen bond with H14. In addition, the elongation of distances between H13, H16, and F25 implies weaker intramolecular interactions of [C4OHMIM][BF_4_] after it adsorbs CHCA. The above phenomena could be explained by the F25···H52 hydrogen bond formation. Therefore, the electronegativity of F25 is decreased and the interaction strengths between F25 and other hydrogen atoms are weaker (see Fig. 3A and Table 1). F23···H13 and F26···C5 distances are also elongated after complex formation, suggesting that the interactions between anion and imidazole ring are weaker as well (Table 1 and Supplementary Table S1). The interactions for [C4NHMIM][BF_4_]-CHCA and [C4OCMIM][BF_4_]-CHCA follow the same trends as [C4OHMIM][BF_4_]-CHCA.

**Table 1:** Bond length (Å) of (A) [C4OHMIM][BF_4_]-CHCA, (B) [C4NHMIM][BF_4_]-CHCA, (C) [C4COOHMIM][BF_4_]_-_CHCA, (D) [C4COOCMIM][BF_4_]-CHCA, and (E) [C4OCMIM][BF_4_]-CHCA.

(A)		(B)		(C)		(D)		(E)	
F23···H13	2.485	F24···H13	2.463	F29···H16	2.341	F32···H13	2.389	F23···H13	2.482
F23···H18	2.414	F24···H18	2.485	F31···H13	2.953	F32···H14	2.698	F23···H18	2.425
F23···H19	2.500	F24···H19	2.533	F31···H14	2.611	F32···H31	3.326	F23···H19	2.496
F25···H13	2.624	F24···H22	2.415	F31···H28	2.987	F32···H57	1.761	F25···H13	2.638
F25···H14	2.786	F24···H31	2.726	F31···H54	1.679	F34···H16	2.443	F25···H14	2.764
F25···H16	2.535	F26···H13	2.763	F33···H19	2.292	F35···H19	2.633	F25···H16	2.530
F25···H52	1.689	F26···H14	2.872	F33···H24	2.519	F35···H24	3.698	F25···H55	1.687
F26···H19	2.389	F26···H16	2.565	F33···C5	2.811	F35···H57	2.630	F26···H19	2.387
F26···C5	2.823	F26···H53	1.688	O26···H13	2.427	F35···C5	2.823	F26···C5	2.829
O50···H13	2.065	F27···H19	2.348	O26···H18	2.260	O26···H13	4.073	O53···H13	2.053
O50···H14	2.447	F27···C5	2.803	O26···H47	2.700	O26···H18	2.423	O53···H14	2.486
		O51···H13	2.060	O52···H13	2.131	O26···H50	2.530		
		O51···H14	2.449	O52···H14	2.395	O26···H52	2.620		
				O53···H28	1.865	O55···H13	1.970		
						O55···H18	2.434		
						O56···H31	2.539		

With the exception of F32···H57, O55···H13, and O55···H18, four more hydrogen bonds are formed when [C4COOCMIM][BF_4_] interacts with CHCA, including F35···H57, O56···H31, O26···H52, and O26···H50, as shown in Fig. 3D and Table 1. As for [C4COOHMIM][BF_4_]-CHCA, five hydrogen bonds (F31···H54, O52···H13, O52···H14, O53···H28, and O26···H47) are formed after interaction (see Fig. 3C and Table 1). Judging from the length of hydrogen bonds, the two dominant hydrogen bonds for [C4COOHMIM][BF_4_]-CHCA are F31···H54 and O53···H28. Distance analysis between the fluorine and hydrogen (carbon) atoms in isolated [C4COOHMIM][BF_4_] and [C4COOHMIM][BF_4_]-CHCA also leads to the conclusion that the interactions between the anion and imidazole ring are weaker upon complex formation.

### Interaction Energies

The interaction energies are important to evaluate the stability of interactions. They are defined as the energy difference between complexes and the sum of isolated ILs and CHCA, and were calculated according to:

1 $${\rm{\Delta }}E={E}_{ILs-CHCA}-({E}_{ILs}+{E}_{CHCA})$$

As shown in Table 2, the interaction energies between CHCA and [C4OHMIM][BF_4_]/[C4NHMIM][BF_4_]/[C4COOHMIM][BF_4_]/[C4COOCMIM][BF_4_]/[C4OCMIM][BF_4_] follow the order of [C4COOCMIM][BF_4_]-CHCA > [C4COOHMIM][BF_4_]-CHCA > [C4OHMIM][BF_4_]-CHCA > [C4OCMIM][BF_4_]-CHCA > [C4NHMIM][BF_4_]-CHCA. Hence, it is deduced that the interaction energies between CHCA and biodegradable ILs with two electronegative atoms are higher than that between CHCA and biodegradable ILs with one electronegative atom. In addition, the interaction energies between CHCA and five types of biodegradable ILs are higher than that between CHCA and [BMIM][BF_4_] (15.22 kcal/mol)^22^, demonstrating that the incorporation of biodegradable groups to ILs promote the extraction of CHCA from crude oil. The interaction energy for [C4COOCMIM][BF_4_]-CHCA is highest among all complexes, which is attributed to the larger number of hydrogen bonding interactions in [C4COOCMIM][BF_4_]-CHCA than other complexes. Similarly, the higher interaction energy of [C4COOHMIM][BF_4_]-CHCA than [C4OHMIM][BF_4_]-CHCA, [C4OCMIM][BF_4_]-CHCA, and [C4NHMIM][BF_4_]-CHCA is ascribed to the larger number of intermolecular hydrogen bonds which is consistent with the geometry analysis.

**Table 2:** Interaction energies between ILs and CHCA.

Complexes	∆E(kcal/mol)
[C4OHMIM][BF_4_]-CHCA	−15.89
[C4NHMIM][BF_4_]-CHCA	−15.81
[C4COOHMIM][BF_4_]_-_CHCA	−16.81
[C4COOCMIM][BF_4_]-CHCA	−17.11
[C4OCMIM][BF_4_]-CHCA	−15.88

### Natural bond orbital (NBO) analyses

To investigate electron distribution and bond characteristics after complex formation, NBO charge distributions of ILs and ILs-CHCA are summarized in Supplementary Table S3. For [C4OHMIM][BF_4_], the charge of H31 (0.46291) is largest among all the hydrogen atoms, which is due to the higher electronegativity of the oxygen atom over that of the carbon atom. In addition, H13 (0.2726) is more positively charged than other hydrogen atoms of the imidazolium ring since it participates in two hydrogen bonding interactions with fluorine atoms. In [C4OHMIM][BF_4_]-CHCA, owing to strong hydrogen bonding of F25···H52, F25 (−0.59611) is more negatively charged than the other fluorine atoms, and H52 (0.52920) is the most positively charged hydrogen atom. The pronounced increase of the charges of H13 (0.28877) and H14 (0.24554) after the formation of complexes is ascribed to the electron transfer from H13 and H14 to O50 because of H13···O50 and H14···O50 interactions. The redistribution of NBO charges for [C4NHMIM][BF_4_]-CHCA and [C4OCMIM][BF_4_]-CHCA are similar to that of [C4OHMIM][BF_4_]-CHCA.

For [C4COOHMIM][BF_4_], the highest charge of H28 (0.51843) is due to the electronegativity of the oxygen atom as well as F31···H28 interaction. Hydrogen bonding between O26···H13 brings about electron transfer from H13 to O26, consequently, the charge of H13 is higher than other hydrogen atoms in the imidazole ring. Moreover, F31···H28, F31···H14, and F31···H13 interactions give rise to the more negative charge of F31 (−0.60678) than other fluorine atoms. With respect to [C4COOHMIM][BF_4_]-CHCA, the charge of H54 (0.53893) is highest among all hydrogen atoms, which results from the strong F31···H54 hydrogen bonding interaction and electron transfer from H54 to adjacent O53. The charge of H28 (0.51321) is second largest because of O53···H28 interaction. In addition, F31 (−0.61088) is far more negatively charged than other fluorine atoms since F31···H54 interactions leads to the electron transfer from H54 to F31. Regarding [C4COOCMIM][BF_4_]-CHCA, F32···H57 interactions induces the charge decrease for F27. Interestingly, the charge of the hydrogen atom (H57) in the carboxylic group of [C4COOCMIM][BF_4_]-CHCA is lower than in the other four complexes. The reason is possibly due to two weaker F32···H57 and F35···H57 hydrogen bonds are formed when [C4COOCMIM][BF_4_] interacts with CHCA, whereas one stronger F ··H hydrogen bond occurs in the other four ILs-CHCA complexes. F32···H57 and F35···H57 hydrogen bond formation could also account for the largest positive charge of H57 among all hydrogen atoms.

The donor-acceptor interactions of ILs-CHCA and their stabilization energy *E*(2) were calculated to determine the extent of interaction. The interaction intensity is reflected by the value of *E*(2). Higher values of *E*(2) indicate that electrons are more likely to migrate from donor to acceptor orbitals and stronger interaction exists between the donor and acceptor. As displayed in Supplementary Table S4, LP(F)-σ*(O-H) has the highest stabilization energy for all these five ILs-CHCA, suggesting that LP(F)-σ*(O-H) is the strongest interaction among all donor-acceptor interactions. In addition, the stabilization energy of LP(O)-σ*(C-H) between ILs and CHCA is substantially higher than most of other donor-acceptor interactions. Except for LP(F)-σ*(C-H) and LP(O)-σ*(C-H) interactions, it is noteworthy that the stabilization energy of LP(O53)-σ*(O27-H28) in [C4COOHMIM][BF_4_]-CHCA is 11.56 kcal/mol, demonstrating that LP(O53) and σ*(O27-H28) interaction is also extraordinarily strong, which is in agreement with geometry analysis and provides further explanation for the higher interaction energies between [C4COOHMIM][BF_4_] and CHCA.

### Topological Properties of Interactions

Through analysis of the values of ρ(r) and Laplacian ∇^2^ρ(r) at the bond critical points (BCPs) of the chemical bonds, the atoms in molecules (AIM) theory^24^ is valuable to characterize chemical bonds, especially hydrogen bonds. To identify bonding interactions for ILs and ILs-CHCA, the results of the AIM analysis is shown in Supplementary Fig. S3 and listed in Supplementary Table S5. The existence of BCPs indicates the formation of hydrogen bonds^25^. [C4OHMIM][BF_4_] and [C4OCMIM][BF_4_] each have 7 BCPs. On the other hand, [C4NHMIM][BF_4_], [C4COOHMIM][BF_4_], and [C4COOCMIM][BF_4_] all have 10 BCPs, suggesting the presence of more intramolecular hydrogen bonds than other ILs, consistent with geometry analysis. Interestingly, three more BCPs are found for [C4OHMIM][BF_4_], [C4NHMIM][BF_4_], and C4OCMIM][BF_4_] after interacting with CHCA, whereas the increase of the number of BCPs are 9 and 5 for [C4COOCMIM][BF_4_] and [C4COOHMIM][BF_4_], respectively. It corresponds to geometry analysis and provides an explanation for the higher interaction energies of [C4COOCMIM][BF_4_]-CHCA and [C4COOHMIM][BF_4_]-CHCA. Additionally, it is deduced that the incorporation of biodegradable groups with two electronegative atoms to ILs form more intermolecular hydrogen bonds with CHCA than those that have one electronegative atom.

As shown in Supplementary Table S5, the value of the Laplacian of the electron density is positive for all ILs, and ILs-CHCA, indicating that the electrons tend to segregate. It also suggests the existence of ionic bonds, hydrogen bonds, and VDW interactions in ILs and ILs-CHCA^22^. Moreover, the strength of interactions can be evaluated by comparing the electron density ρ(r) value of BCPs. Larger values of ρ(r) correspond to stronger interactions^26^. The electron densities of O26···H13 and F31···H28 in [C4COOHMIM][BF_4_] are larger than the electron densities of other ILs, which indicates the presence of stronger hydrogen bonds. In addition, the electron density of F25···H52 is greatest for [C4OHMIM][BF_4_]-CHCA, implying that the hydrogen bonds between fluorine and hydrogen is the strongest hydrogen bond. Further analysis of the largest electron density for other ILs-CHCA also demonstrates that the F···H interaction is the strongest interaction. Compared to the distances of other hydrogen bonds, the F···H distance is shortest for all five ILs-CHCA, therefore, it is inferred that electron density is correlated to intermolecular hydrogen bond distances. The electron density of O-H ranks second for [C4NHMIM][BF_4_]-CHCA and [C4OCMIM][BF_4_]-CHCA, which suggests that O-H hydrogen bond is also critical in the interaction between ILs and CHCA. Except for hydrogen bonding interactions, the F35···O55 and F35···O26 pairs in [C4COOCMIM][BF_4_]-CHCA indicate the existence of anion-anion interactions.

### Noncovalent interaction (NCI) analyses

**Figure 4:**
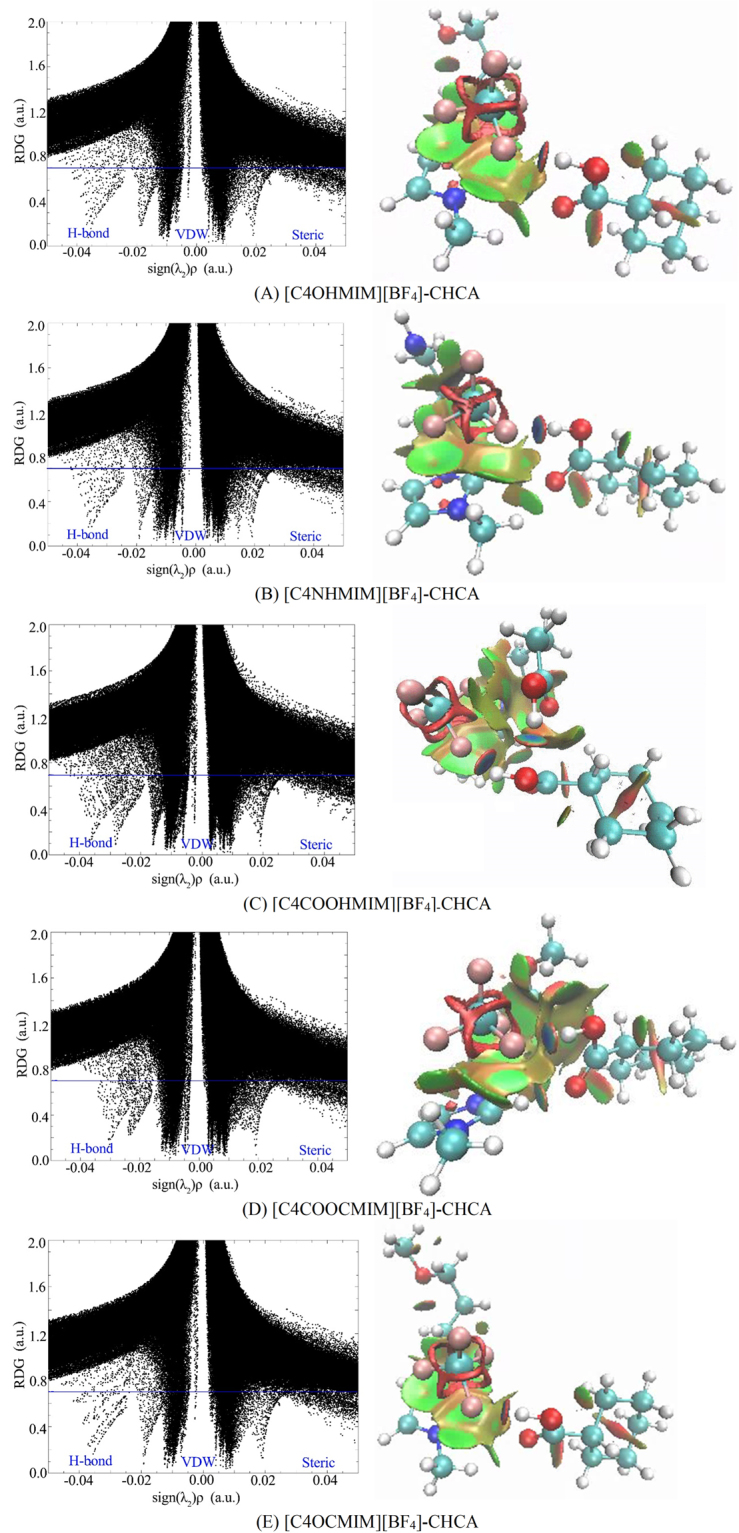
The sign(λ_2_)ρ versus RDG (left) and the gradient isosurfaces (right) for (**A**) [C4OHMIM][BF_4_]-CHCA, (**B**) [C4NHMIM][BF_4_]-CHCA, (**C**) [C4COOHMIM][BF_4_]-CHCA, (**D**) [C4COOCMIM][BF_4_]-CHCA, and (E) [C4OCMIM][BF_4_]-CHCA. Note: red indicates sign(λ_2_)ρ > 0 and blue indicates sign(λ_2_)ρ < 0.

Through performing NCI analyses, which is based on the reduced density gradient (RDG)^27^, the intramolecular and intermolecular interaction types and strengths can be evaluated^28^. In the plots of RDG versus sign(λ_2_)ρ, the peaks in the sign(λ_2_)ρ < 0, sign(λ_2_)ρ = 0, and sign(λ_2_)ρ > 0 region suggest attractive interactions, VDW interactions, and steric effects, respectively. Furthermore, the interaction types and strength can be identified through analyzing the color and area in the gradient isosurface diagram; red indicates steric repulsions, green means weak interactions such as VDW interactions, and blue represents strong attractive interactions such as hydrogen bonds^29,30^. To investigate intramolecular and intermolecular interaction types and strength, the plots of RDG versus sign(λ_2_)ρ and the gradient isosurface (s = 0.6 a.u.) for ILs are shown in Supplementary Fig. S4. In addition, the plots of RDG versus sign(λ_2_)ρ and gradient isosurface (s = 0.7 a.u.) for ILs-CHCA are displayed in Fig. 4.

The results in Supplementary Fig. S4 reveals that there is no peak in the sign(λ_2_)ρ < 0 region for [C4OHMIM][BF_4_], [C4NHMIM][BF_4_], and [C4OCMIM][BF_4_], meaning that no apparent intramolecular hydrogen bonds exists in those ILs. Whereas prominent peaks at −0.02 a.u. in Supplementary Fig. S4C corresponds to the F31···H28, O26···H18, and O26···H13 hydrogen bonds in [C4COOHMIM][BF_4_], which is consistent with geometry analysis, NBO analysis, and AIM analysis. The peaks at −0.02 a.u. for [C4COOCMIM][BF_4_] is not as strong as those for [C4COOHMIM][BF_4_], which is attributed to the smaller electron densities of intramolecular hydrogen bonds for [C4COOCMIM][BF_4_]. On the other hand, the presence of peaks at 0.02 a.u., shown in Supplementary Fig. S4B and D, represents strong steric intramolecular interactions for [C4NHMIM][BF_4_] and [C4COOCMIM][BF_4_]. Compared to the color and area of gradient isosurface for [BMIM][BF_4_], the gradient isosurface analysis of five biodegradable ILs imply that the addition of biodegradable groups do not destroy the LP-π interactions between the anion and cation. On the other hand, the area of LP-π interactions are larger for [C4NHMIM][BF_4_], [C4COOHMIM][BF_4_], and [C4COOCMIM][BF_4_] over that of [C4OHMIM][BF_4_] and [C4OCMIM][BF_4_].

After interacting with CHCA, all the ILs-CHCA complexes have spikes at 0.02 a.u., as shown in Fig. 4, indicating the existence of steric effects for all these interactions. [C4OHMIM][BF_4_]-CHCA, [C4NHMIM][BF_4_]-CHCA, and [C4OCMIM][BF_4_]-CHCA have spikes at −0.04 a.u., which corresponds to the dark blue circle between the fluorine atom and the hydrogen atom, further confirming the existence of strong hydrogen bonds. The peaks at −0.02 a.u. in Fig. 4A,B and E denote hydrogen bonds formed between oxygen and hydrogen atoms for [C4OHMIM][BF_4_]-CHCA, [C4NHMIM][BF_4_]-CHCA, and [C4OCMIM][BF_4_]-CHCA. According to AIM analysis, the electron density of O55···H13 in [C4COOCMIM][BF_4_]-CHCA is larger than the electron density of O···H in [C4OHMIM][BF_4_]-CHCA, [C4NHMIM][BF_4_]-CHCA, and [C4OCMIM][BF_4_]-CHCA, which can account for the deviation of the peaks from −0.2 a.u. (see Fig. 4D and Supplementary Table S5). On the other hand, the highest electron densities of F34···H16 for [C4COOCMIM][BF_4_]-CHCA are smaller than the highest electron densities of F···H in other complexes, hence, the strongest peak for [C4COOCMIM][BF_4_]-CHCA is located further from −0.4a.u., as shown in Fig. 4D and Supplementary Table S5. In addition, there are three spikes for [C4COOHMIM][BF_4_]-CHCA in sign(λ_2_)ρ < 0 region, and can be explained by the strong electron densities of: F31···H54, O53-H28, and O26···H18 in AIM analysis (see Fig. 4C and Supplementary Table S5).

### Electron Density Difference Analysis

When ILs interact with CHCA, there is a transfer of electron density during the interaction process^31^. The electron density change was determined by subtracting the electron density of complexes from the sum of electron density of isolated ILs and CHCA:

2 $${\rm{\Delta }}\rho ={\rho }_{ILs-CHCA}-({\rho }_{ILs}+{\rho }_{CHCA})$$

To evaluate electron density redistribution caused by the interaction between ILs and CHCA, electron density distribution maps were plotted. As depicted in Supplementary Fig. S5, the obvious electron density change mostly locates around the interaction region between ILs and CHCA. The formation of F25···H52 hydrogen bonds in [C4OHMIM][BF_4_]-CHCA, shown in Supplementary Fig. S5A, increases the electron density of F25 and decreases that of H52 because of electron transfer from H52 to F25. In addition, due to O50···H13 and O50···H14 interactions, the electron density of O50 increases whereas the electron densities of H13 and H14 decrease. The formation of hydrogen bonds between electronegative and hydrogen atoms provoke the increase of density for the electronegative atom such as F and O, and induce the decrease of electron density for hydrogen atom.

In summary, the interaction energy between biodegradable ILs and CHCA is higher than that between [BMIM][BF_4_] and CHCA. Moreover, biodegradable ILs with two electronegative atoms have higher interaction energy with CHCA than that having one electronegative atom. Compared with the extraction mechanism for [BMIM][BF_4_], the main interaction is still hydrogen bonding. However, biodegradable ILs form more hydrogen bonds with CHCA than [BMIM][BF_4_]. Therefore, it is deduced that the design of biodegradable ILs promote the extraction of CHCA. Additionally, the greater the number of electronegative atoms in biodegradable group of ILs, the easier it is to extract CHCA.

## Computational Methods

The density functional computations were carried out using Gaussian 09 program packages^32^. The M06–2X functional is suitable for calculation with nonmetals and is recommended to calculate main-group thermochemistry, kinetics, NCI, and electronic excitation energies to valence and Rydberg states^33^. It also has better performance than B3LYP and PW91 for systems with dispersion and ionic hydrogen-bonding interactions^34^ and is compatible to CCSD(T) and MP2 in describing NCI^35^. The geometries of CHCA and ILs with biodegradable groups were fully optimized by the M06–2X method in combination with the empirical dispersion-correction (DFT-D3)^36^ method and the 6–311 +  + G(d,p) basis set. The ILs-CHCA interaction structures were also optimized by employing the same method and basis set. Vibrational analyses were performed to confirm that the structures are at minimal energy without imaginary frequencies. The interaction energies were calculated with the correction by the counterpoise method for basis set superposition error^37^. The second-order perturbation energy *E*(2) in NBO was determined by using the Gaussian 09 program packages with the M06–2X/6–311 +  + G(d,p) level of theory. The Multiwfn software package was adopted to analyze the wave functions of the optimized structures to obtain NCI, and electron density differences^38,39^. The interaction regions of NCI analysis were visualized and colored with the Visual Molecular Dynamics (VMD) software package^40^. Topological properties were analyzed by using AIM theory^24^.

## Electronic supplementary material

Supplementary Information
