# Supplementary material for: Interactions of Biodegradable Ionic Liquids with a Model Naphthenic Acid
Source: Sci Rep. 2018 Jan 9;8:176. doi: 10.1038/s41598-017-18587-1 (PMC5760565; doi:10.1038/s41598-017-18587-1)
Supplement: Supplementary file 1 — Supplementary Information [file 41598_2017_18587_MOESM1_ESM.doc]

Supporting information for

**Interactions of Biodegradable Ionic Liquids with a
Model Naphthenic Acid**

Chongchong Wu^a^, Alex De Visscher^a, b^, Ian D. Gates^a*^

^a^Department of Chemical and Petroleum Engineering, University of Calgary, Alberta, Canada

^b^Department of Chemical and Materials Engineering, Concordia University, Quebec, Canada

*Corresponding Author: Ian Gates

Phone: +1-403-220-5752

Email: [ian.gates@ucalgary.ca](mailto:ian.gates@ucalgary.ca)

| 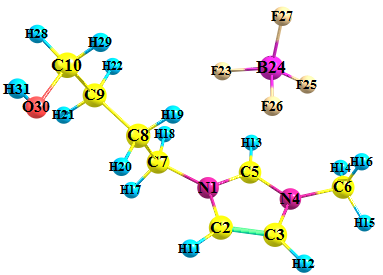 | 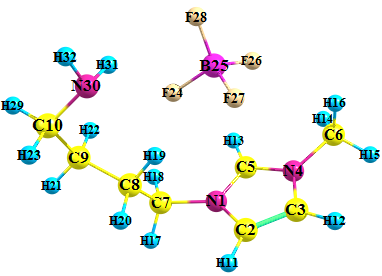 |
| --- | --- |
| (A) [C4OHMIM][BF_4_] | (B) [C4NHMIM][BF_4_] |
| 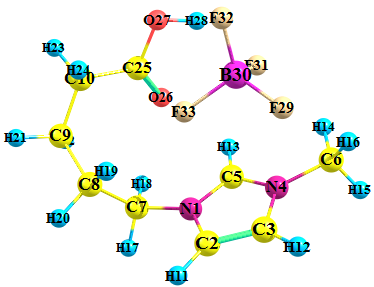 | 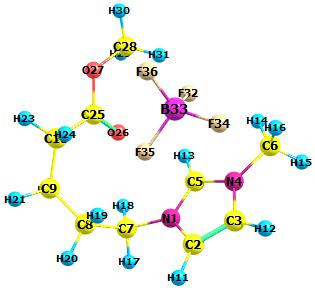 |
| (C) [C4COOHMIM][BF_4_] | (D) [C4COOCMIM][BF_4_] |
| 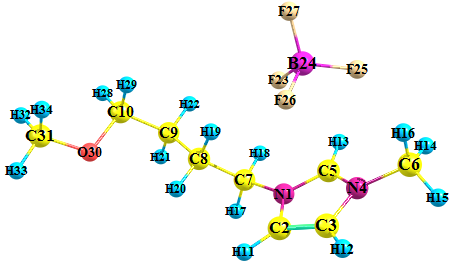 |  |
| (E) [C4OCMIM][BF_4_] |  |

Supplementary Figure S1. The optimized structures of (A) [C4OHMIM][BF_4_], (B) [C4NHMIM][BF_4_], (C) [C4COOHMIM][BF_4_], (D) [C4COOCMIM][BF_4_], and (E) [C4OCMIM][BF_4_].

| 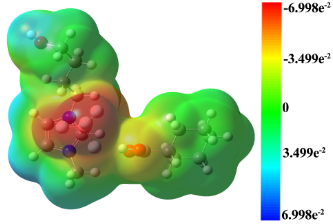 | 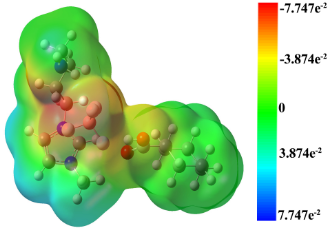 |
| --- | --- |
| (A) [C4OHMIM][BF_4_]-CHCA | (B) [C4NHMIM][BF_4_]-CHCA |
| 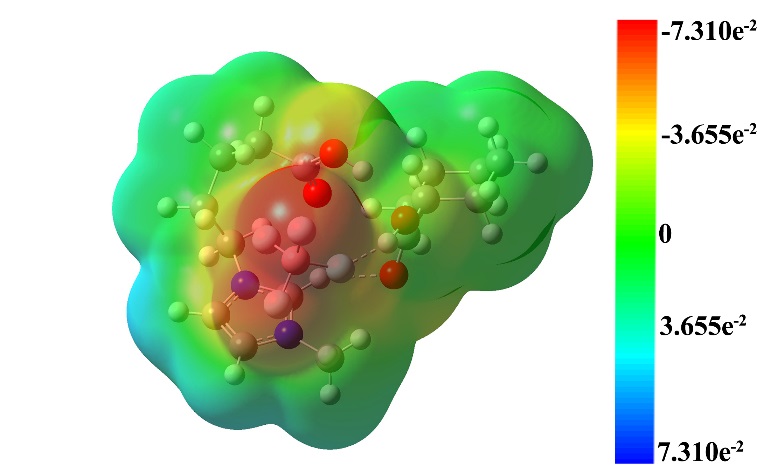 | 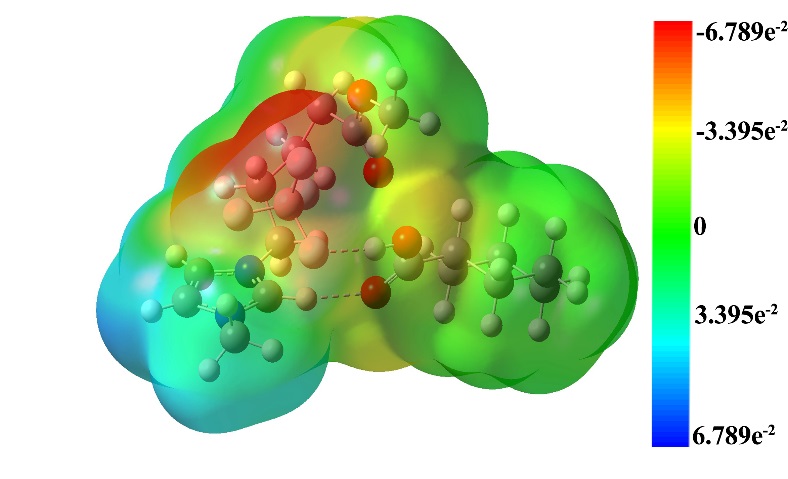 |
| (C) [C4COOHMIM][BF_4_]-CHCA | (D) [C4COOCMIM][BF_4_]-CHCA |
| 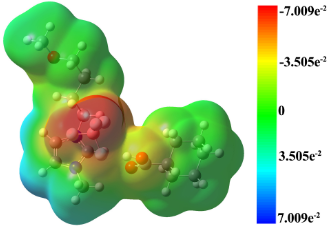 |  |
| (E) [C4OCMIM][BF_4_]-CHCA |  |

Supplementary Figure S2. The electrostatic potential (a.u.) of (A)[C4OHMIM][BF_4_]-CHCA, (B) [C4NHMIM][BF_4_]-CHCA, (C) [C4COOHMIM][BF_4_]-CHCA, (D) [C4COOCMIM][BF_4_]-CHCA, and (E) [C4OCMIM][BF_4_]-CHCA.

| 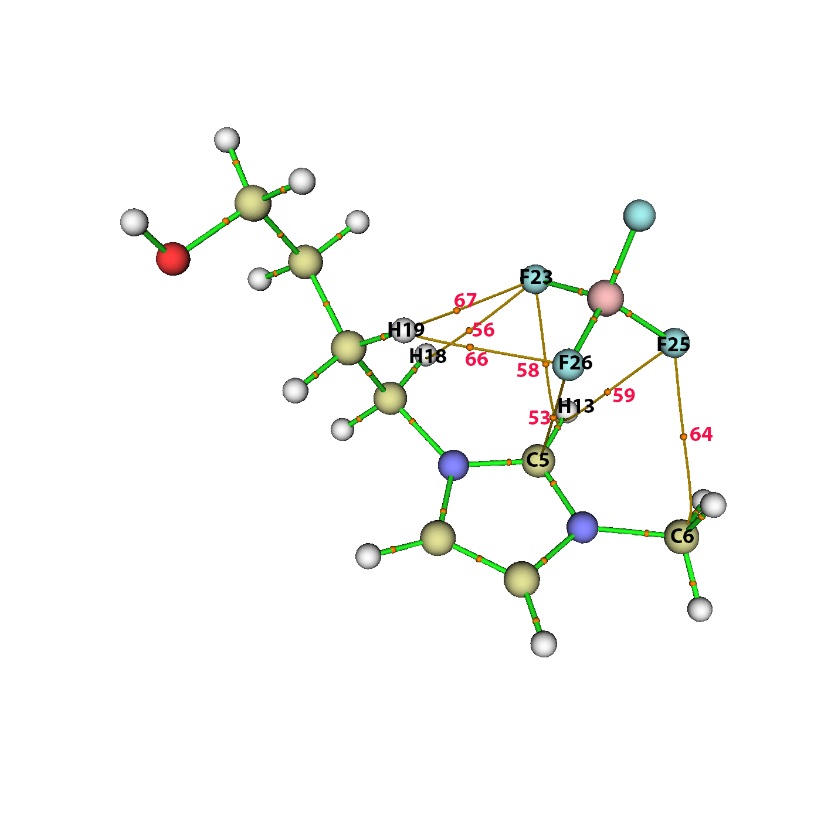 | 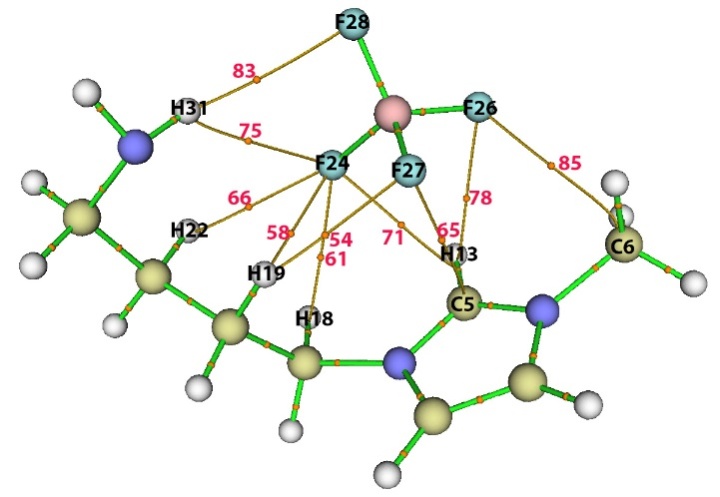 |
| --- | --- |
| (A) [C4OHMIM][BF_4_] | (B) [C4NHMIM][BF_4_] |
| 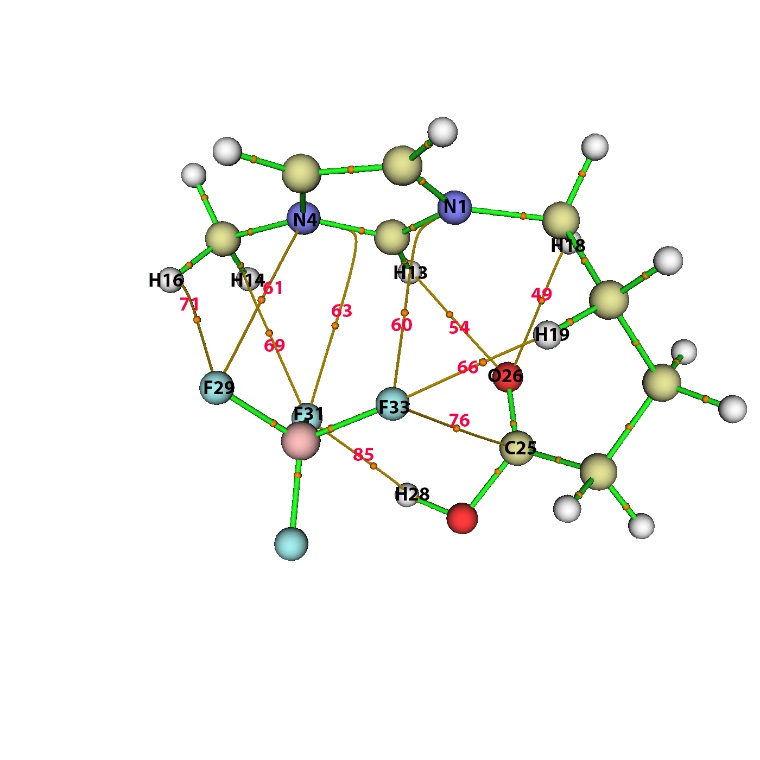 | 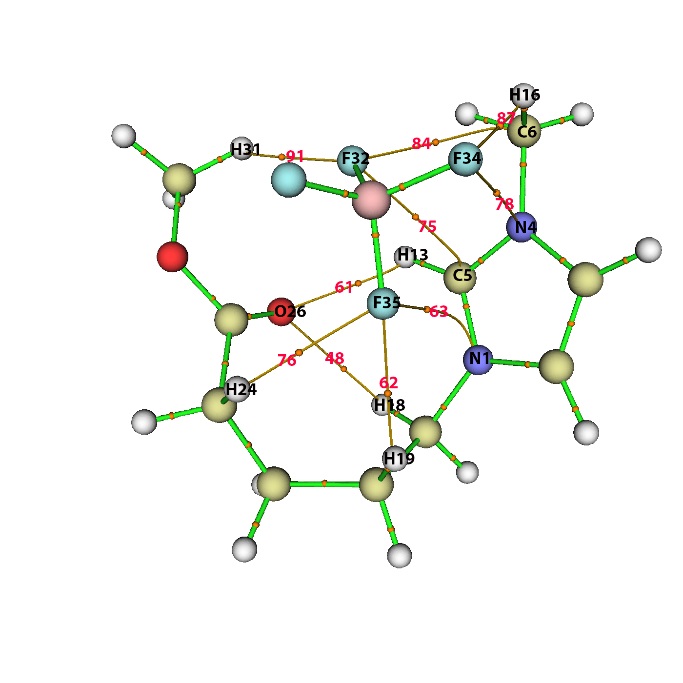 |
| (C) [C4COOHMIM][BF_4_] | (D) [C4COOCMIM][BF_4_] |
| 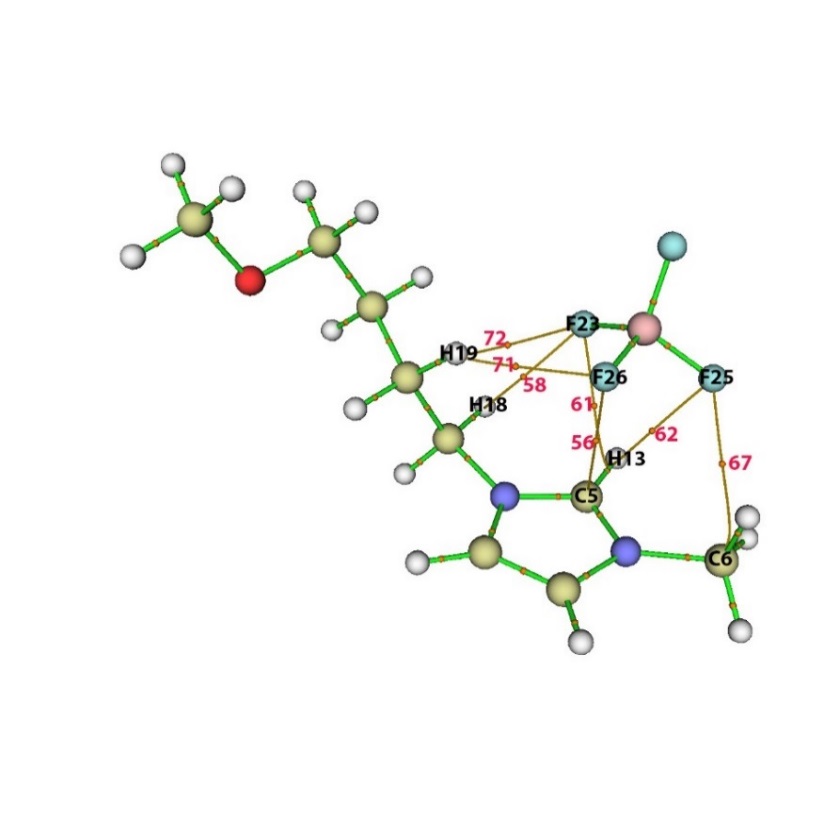 | 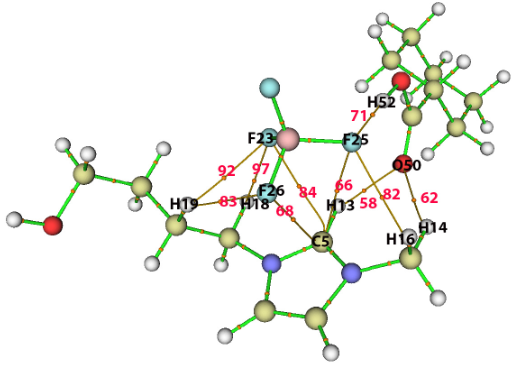 |
| (E) [C4OCMIM][BF_4_] | (F) [C4OHMIM][BF_4_]-CHCA |
| 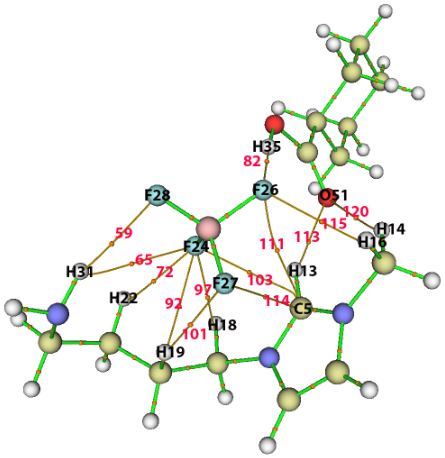 | 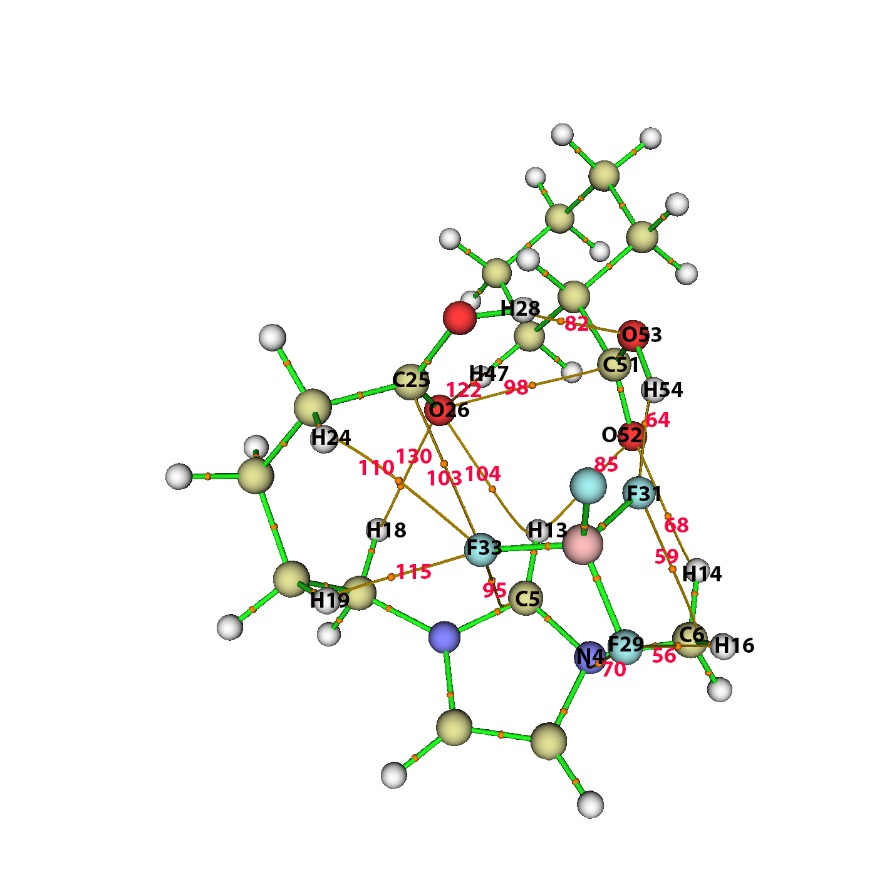 |
| (G) [C4NHMIM][BF4]-CHCA | (H) [C4COOHMIM][BF_4_]-CHCA |
| 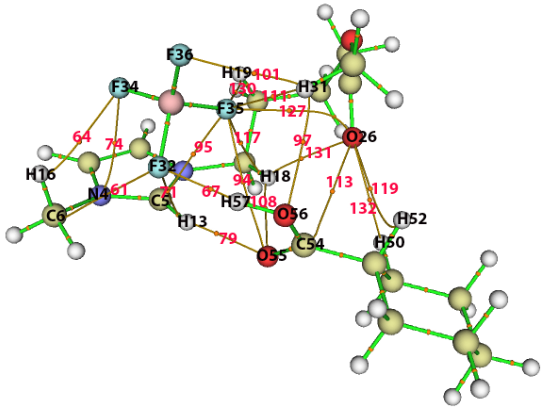 | 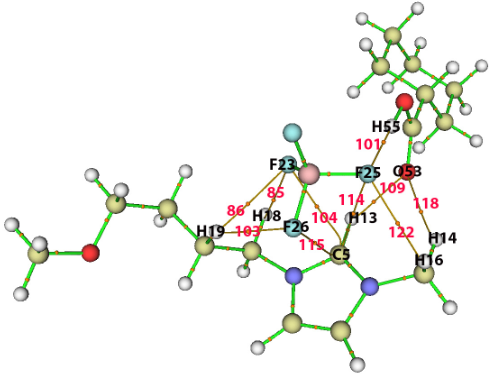 |
| (I) [C4COOCMIM][BF_4_]-CHCA | (J) [C4OCMIM][BF_4_]-CHCA |

Supplementary Figure S3. BCPs and bond paths in (A) [C4OHMIM][BF_4_], (B) [C4NHMIM][BF_4_], (C) [C4COOHMIM][BF_4_], (D) [C4COOCMIM][BF_4_], (E) [C4OCMIM][BF_4_], (F) [C4OHMIM][BF_4_]-CHCA, (G) [C4NHMIM][BF_4_]-CHCA, (H) [C4COOHMIM][BF_4_]-CHCA, (I) [C4COOCMIM][BF_4_]-CHCA, and (J) [C4OCMIM][BF_4_]-CHCA.

| 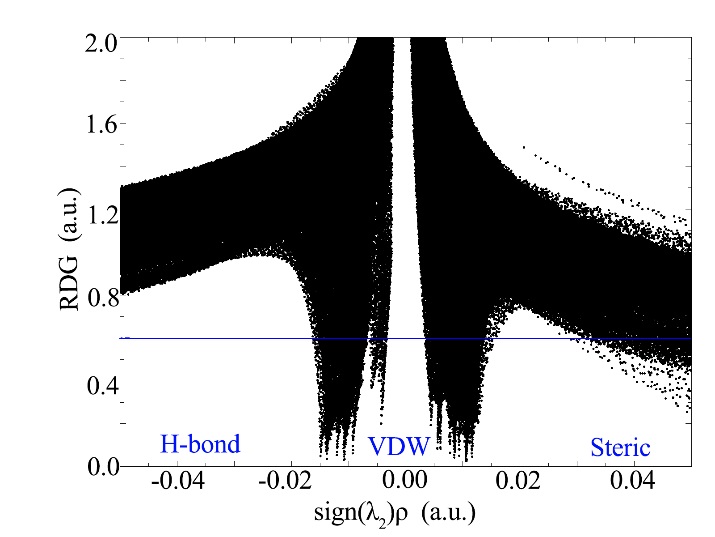 | 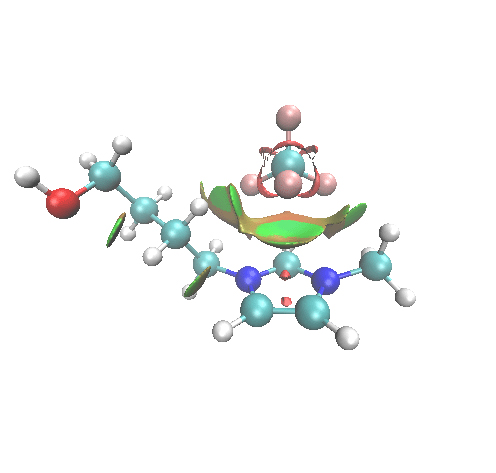 |
| --- | --- |
| (A) [C4OHMIM][BF_4_] | |
| 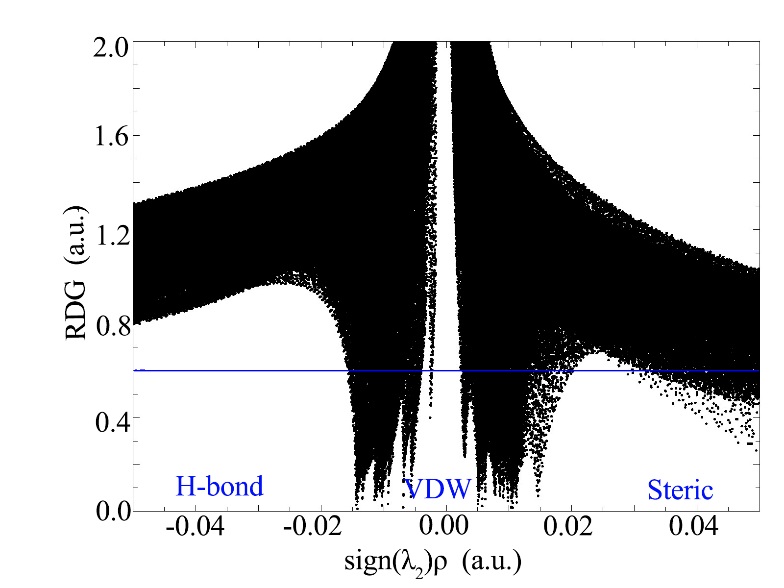 | 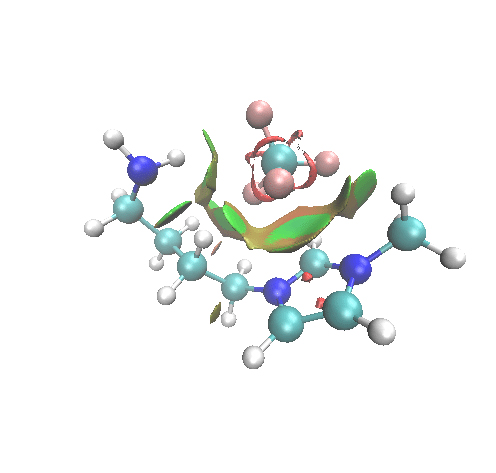 |
| (B) [C4NHMIM][BF_4_] | |
| 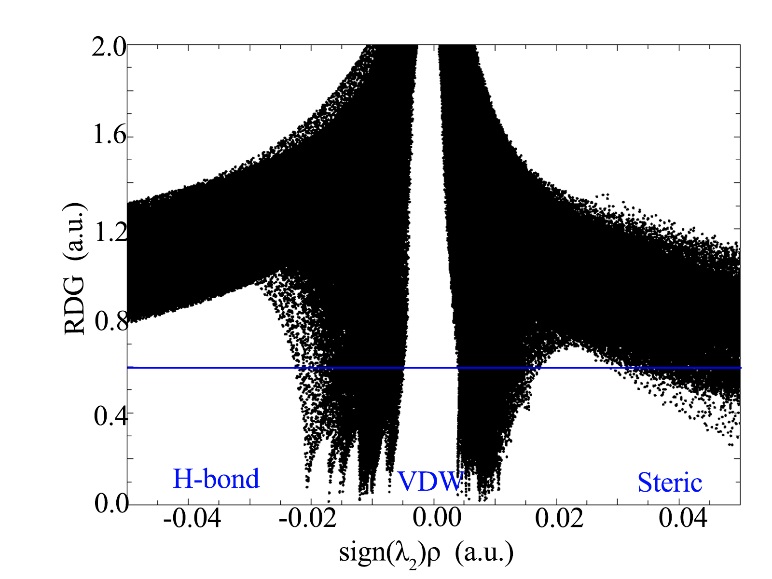 | 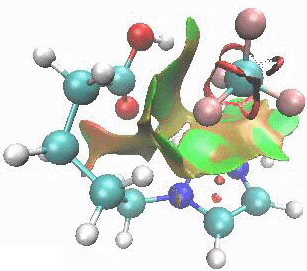 |
| (C) [C4COOHMIM][BF_4_] | |
| 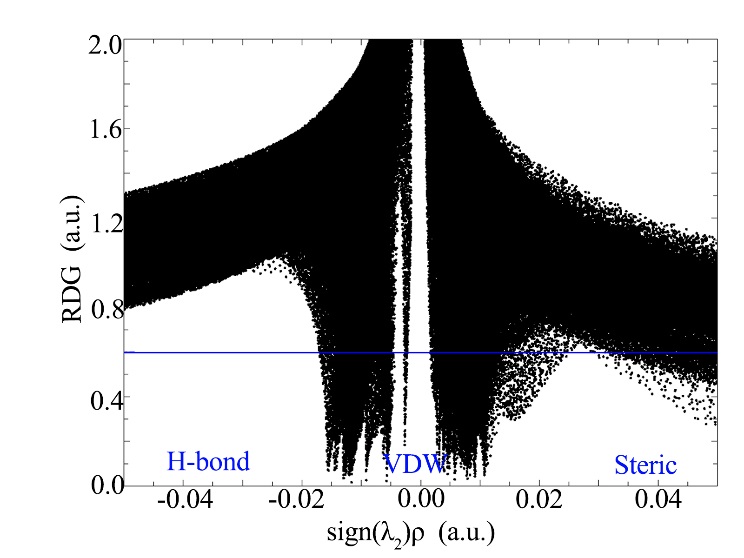 | 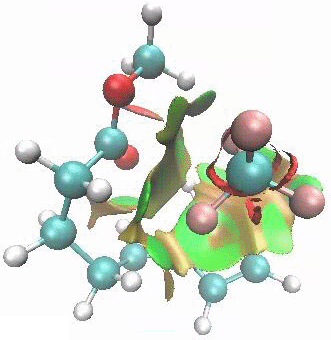 |
| (D) [C4COOCMIM][BF_4_] | |
| 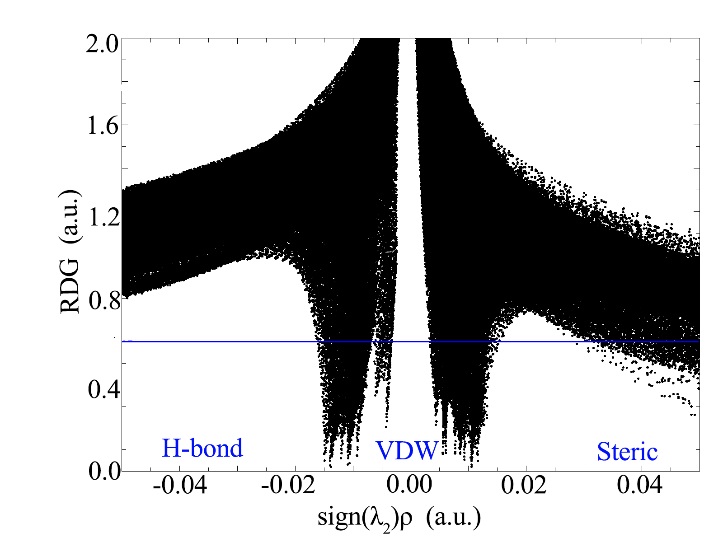 | 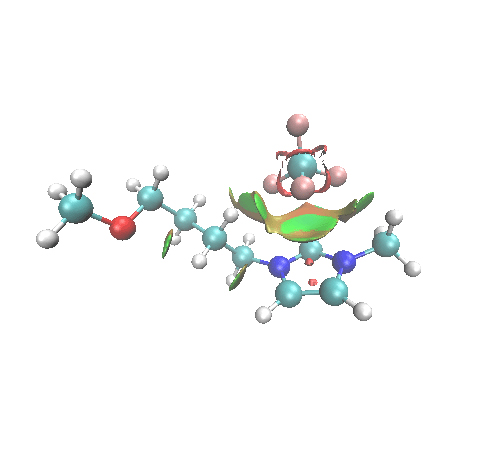 |
| (E) [C4OCMIM][BF_4_] | |

Supplementary Figure S4. The sign(λ2)ρ vs RDG (left) and the gradient isosurfaces (right) for (A) [C4OHMIM][BF_4_], (B) [C4NHMIM][BF_4_], (C) [C4COOHMIM][BF_4_], (D) [C4COOCMIM][BF_4_], and (E) [C4OCMIM][BF_4_]. Note: red indicatessign(λ_2_)ρ>0 and blue indicatessign(λ_2_)ρ<0.

| 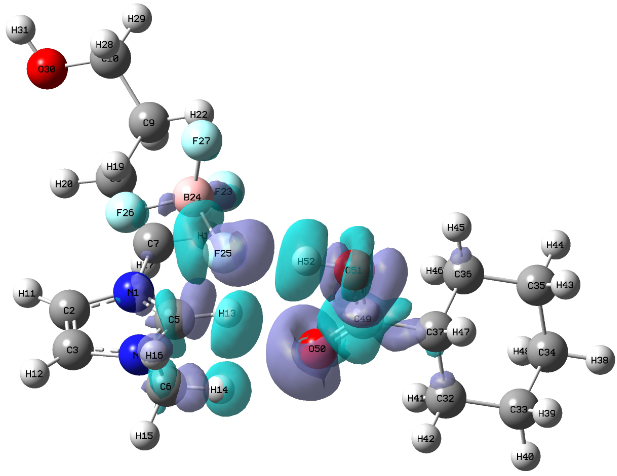 |
| --- |
| (A) [C4OHMIM][BF_4_]-CHCA |
| 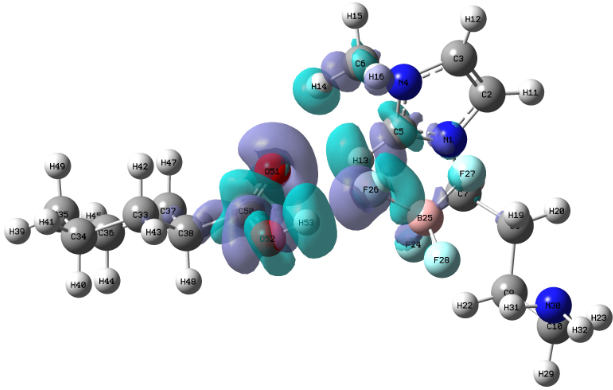 |
| (B) [C4NHMIM][BF_4_]-CHCA |
| 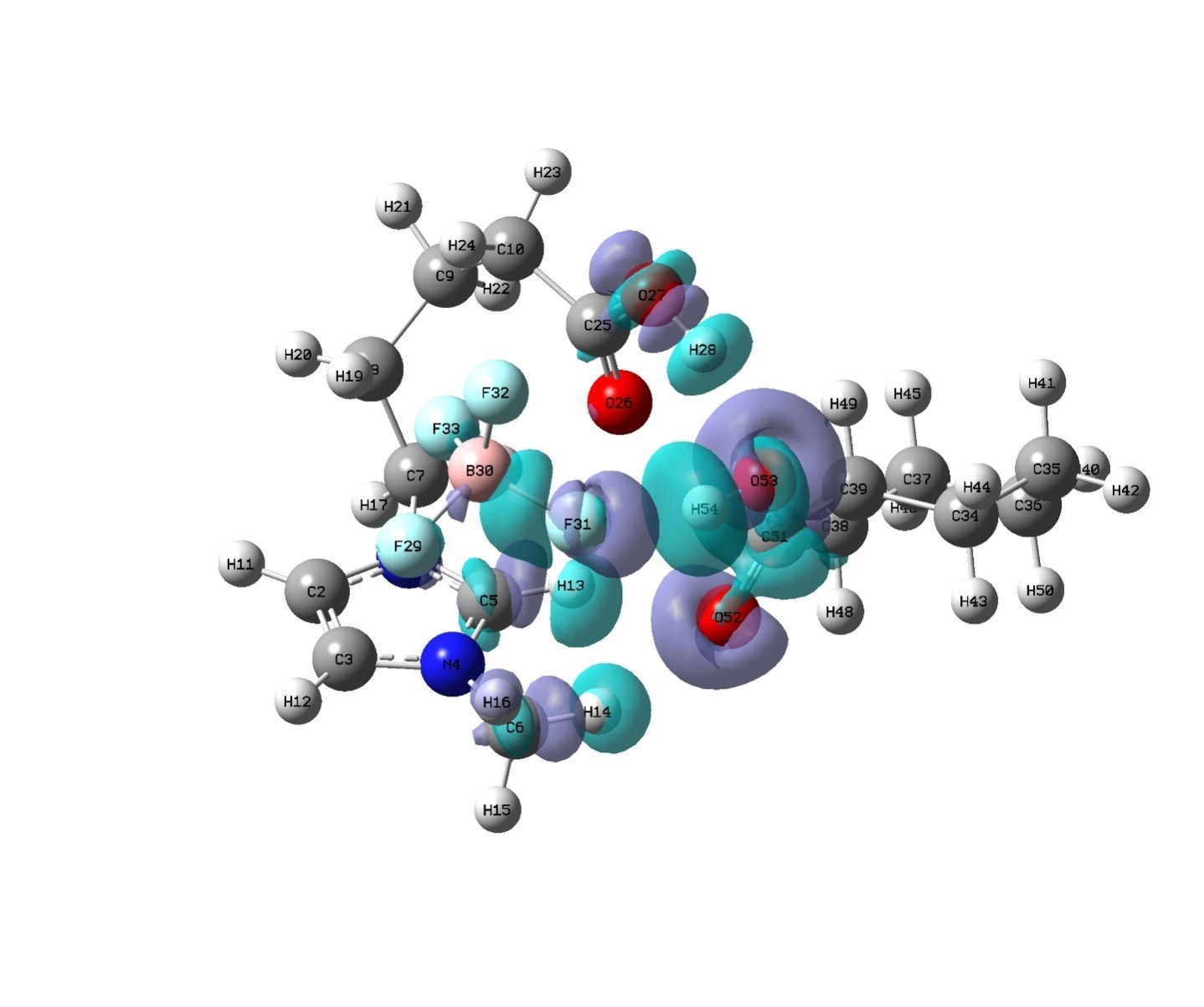 |
| (C) [C4COOHMIM][BF_4_]-CHCA |
| 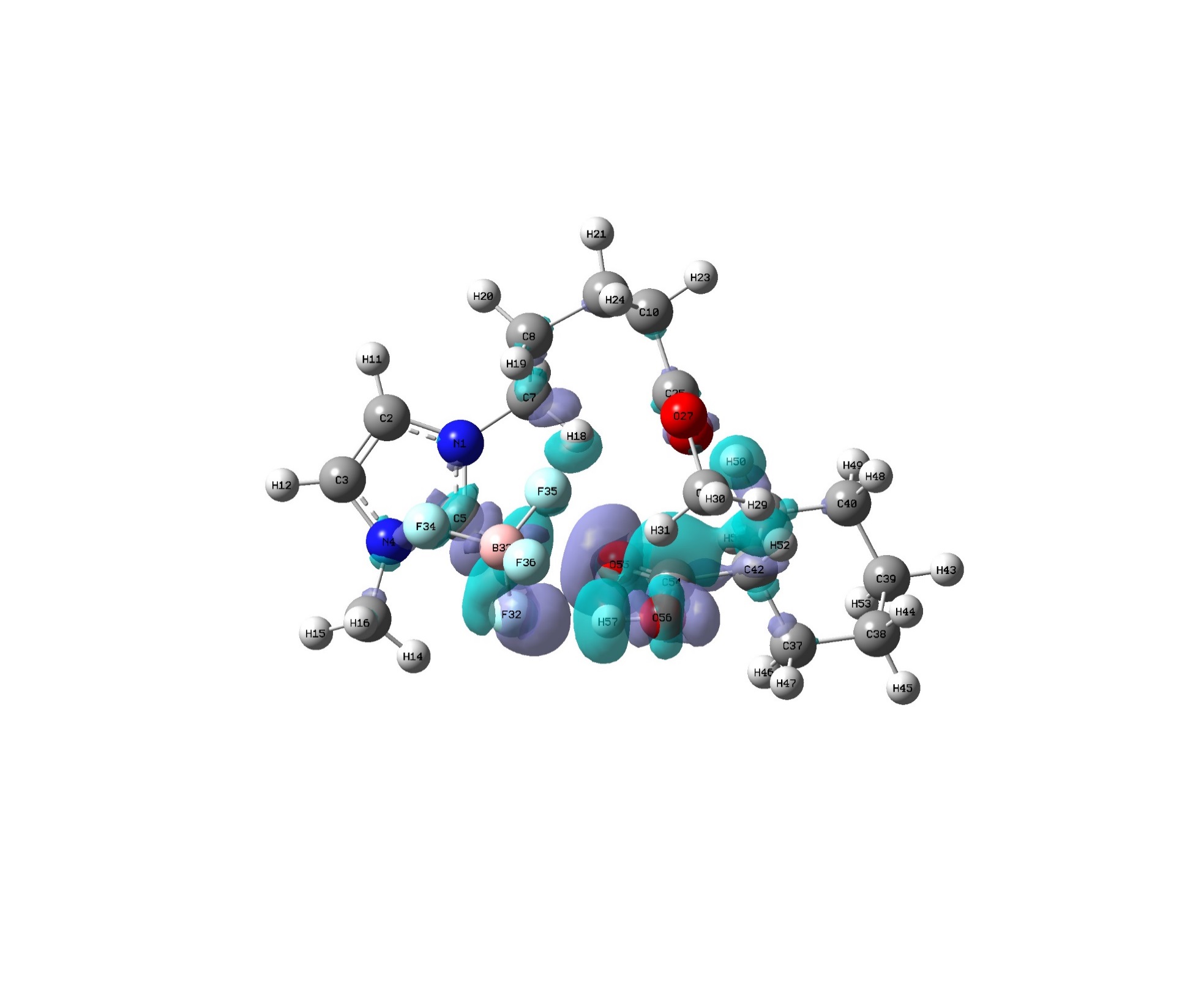 |
| (D) [C4COOCMIM][BF_4_]-CHCA |
| 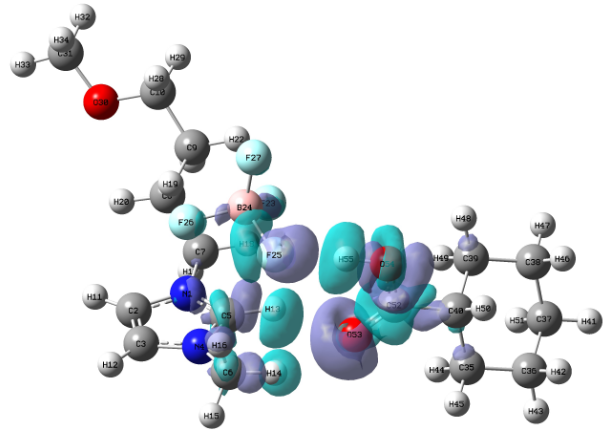 |
| (E) [C4OCMIM][BF_4_]-CHCA |

Supplementary Figure S5. The different electron densities of five types of ionic liquids and model NAs. Note: the purple and green isosurfaces (0.001 a.u.) represent the region inwhich electron density is increased and decreased after interaction between cation and anion of ionic liquids and model NAs, respectively.(A) [C4OHMIM][BF_4_]-CHCA,(B) [C4NHMIM][BF_4_]-CHCA, (C) [C4COOHMIM][BF_4_]-CHCA, (D) [C4COOCMIM][BF_4_]-CHCA, and (E) [C4OCMIM][BF_4_]-CHCA.

Supplementary Table S1. Bond length (Å) of (A) [C4OHMIM][BF_4_], (B) [C4NHMIM][BF_4_], (C) [C4COOHMIM][BF_4_], (D) [C4COOCMIM][BF_4_], and (E) [C4OCMIM][BF_4_].

| (A) | | (B) | | (C) | | (D) | | (E) | |
| --- | --- | --- | --- | --- | --- | --- | --- | --- | --- |
| F23···H13 | 2.346 | F24···H13 | 2.340 | F29···H16 | 2.222 | F32···H13 | 2.615 | F23···H13 | 2.346 |
| F23···H18 | 2.379 | F24···H18 | 2.447 | F31···H13 | 2.710 | F32···H14 | 2.526 | F23···H18 | 2.378 |
| F23···H19 | 2.441 | F24···H19 | 2.462 | F31···H14 | 2.465 | F32···H31 | 2.197 | F23···H19 | 2.447 |
| F25···H13 | 2.295 | F24···H22 | 2.398 | F31···H28 | 1.994 | F34···H16 | 2.215 | F25···H13 | 2.297 |
| F25···H14 | 2.606 | F24···H31 | 2.526 | F33···H19 | 2.275 | F35···H19 | 2.252 | F25···H14 | 2.610 |
| F25···H16 | 2.531 | F26···H13 | 2.335 | F33···H24 | 2.676 | F35···H24 | 2.316 | F25···H16 | 2.526 |
| F26···H19 | 2.413 | F26···H14 | 2.631 | F33···C5 | 2.816 | F35···C5 | 2.783 | F26···H19 | 2.412 |
| F26···C5 | 2.728 | F26···H16 | 2.546 | O26···H13 | 2.164 | O26···H13 | 2.185 | F26···C5 | 2.731 |
| O30···H20 | 2.528 | F27···H19 | 2.405 | O26···H18 | 2.307 | O26···H18 | 2.279 | O30···H21 | 2.614 |
| O30···H21 | 2.601 | F27···C5 | 2.712 |  |  |  |  | O30···H28 | 2.063 |
| O30···H28 | 2.078 | N30···H19 | 2.417 |  |  |  |  | O30···H29 | 2.065 |
| O30···H29 | 2.081 | N30···H22 | 2.728 |  |  |  |  | O30···H32 | 2.073 |
|  |  | N30···H23 | 2.080 |  |  |  |  | O30···H33 | 2.022 |
|  |  | N30···H29 | 2.154 |  |  |  |  | O30···H34 | 2.072 |

Supplementary Table S2. The Cartesian coordinates of (A) [C4OHMIM][BF4]-CHCA, (B) [C4NHMIM][BF_4_]-CHCA, (C) [C4COOHMIM][BF_4_]-CHCA, (D) [C4COOCMIM][BF_4_]-CHCA, and (E) [C4OCMIM][BF_4_]-CHCA.

| (A) [C4OHMIM][BF4]-CHCA | | | | (B) [C4NHMIM][BF_4_]-CHCA | | | |
| --- | --- | --- | --- | --- | --- | --- | --- |
| N  C  C  N  C  C  C  C  C  C  H  H  H  H  H  H  H  H  H  H  H  H  F  B  F  F  F  H  H  O  H  C  C  C  C  C  C  H  H  H  H  H  H  H  H  H  H  H  C  O  O  H | -2.06674600  -3.02533300  -2.45830700  -1.16452500  -0.95773200  -0.21832100  -2.27412900  -3.45753800  -3.44592200  -4.66074100  -4.01922300  -2.86080600  -0.03819200  0.75724600  -0.18424900  -0.53850100  -2.41146700  -1.35505000  -3.38582700  -4.39876900  -3.42801900  -2.54346300  -1.03764200  -1.34602600  -0.22562200  -2.46361400  -1.52758000  -4.70038900  -4.59251600  -5.81409800  -6.59833300  4.64108200  6.00857400  5.91018500  5.28864900  3.92056500  4.02672300  6.89778200  6.70271300  6.42414300  3.96232800  4.72127300  5.95400500  5.19113100  3.49995900  3.22524800  4.65449000  5.28706200  2.65721000  1.99325700  2.24110800  1.32463700 | -0.73743500  -1.71136300  -2.89148500  -2.61577100  -1.30784200  -3.55094600  0.71353800  1.15533700  2.66839700  3.14173500  -1.48080700  -3.88889600  -0.81244500  -3.06950400  -4.45869600  -3.76171600  0.97134000  1.17250500  0.65536600  0.85843000  3.18262600  2.95320000  0.53526200  -0.58371100  -1.48772200  -1.23946100  -0.22175400  2.62915100  4.22017500  2.83862400  3.07104700  -0.25799400  0.40575100  1.92416700  2.29180500  1.63213700  0.10360400  2.38213200  0.00792000  0.15412600  0.07582900  -1.34387200  1.96171100  3.37645800  1.86510800  2.01883000  -0.27917900  2.33140200  -0.50631000  -0.96118500  -0.46779800  -0.81849600 | -1.49762300  -1.66997100  -1.31356800  -0.93256100  -1.03949400  -0.32856000  -1.59973400  -0.74735000  -0.55257400  0.22234600  -2.01092300  -1.28441200  -0.76218500  -0.30597900  -0.92913800  0.69059900  -2.65256700  -1.23584100  0.22110200  -1.21760400  -1.52002300  -0.00288900  1.07125300  1.87976900  1.76618600  1.34753800  3.18898100  1.19099200  0.40321400  -0.55344000  -0.05246400  -1.03782400  -1.20991600  -1.04743300  0.30131600  0.48202800  0.31299500  -1.14682700  -0.45948400  -2.18900700  -1.82969900  -1.13339600  1.10815000  0.39477400  1.46218300  -0.27349700  1.12671500  -1.85298500  0.48894900  -0.42206300  1.74272600  1.80297200 | N  C  C  N  C  C  C  C  C  C  H  H  H  H  H  H  H  H  H  H  H  H  H  F  B  F  F  F  H  N  H  H  C  C  C  C  C  C  H  H  H  H  H  H  H  H  H  H  H  C  O  O  H | 2.17452000  3.04502800  2.40597600  1.15690500  1.04826600  0.18367600  2.50414000  3.68340300  3.54698800  4.58113100  4.03822200  2.73164300  0.18627200  -0.75338700  0.05217700  0.54777500  2.69183000  1.60769900  3.70670500  4.62987600  3.62860400  2.54915200  5.58440300  1.38032100  1.64392600  0.42164600  2.60602000  2.02295200  4.38975000  4.54618500  3.60846600  5.15264500  -4.80070700  -6.19910400  -6.60587100  -5.57559900  -4.17668200  -3.77003300  -7.59487800  -6.20755300  -6.92254800  -4.80482500  -4.50078400  -5.56391700  -5.85928700  -3.44157000  -4.15896000  -3.71693800  -6.68189400  -2.40299800  -1.81424800  -1.92369300  -1.04219600 | 1.39385000  2.41539700  3.22909400  2.68967700  1.57519300  3.11669100  0.17144800  -0.56131300  -2.07704800  -2.83203500  2.46081800  4.12407600  0.92342100  2.60233200  4.19545700  2.82254800  0.44847300  -0.44688500  -0.31457200  -0.22284500  -2.38658500  -2.36021800  -2.64120900  -0.98024100  -0.37808700  0.26077000  0.62265100  -1.33234000  -3.90878800  -2.36475000  -2.40702800  -2.91421600  -0.30831100  -0.79853900  -0.30239000  -0.71589700  -0.22070800  -0.72532600  -0.68574700  -1.89509900  -0.46978200  0.78657600  -0.70031300  -1.80966200  -0.32889400  -0.53818300  0.87431300  -1.82094400  0.79165100  -0.22475700  0.67404200  -0.85147500  -0.48680100 | -1.37227300  -1.06374500  -0.18567500  0.02403800  -0.69108200  1.02678800  -2.12167100  -1.47617000  -1.61608300  -0.78285300  -1.47517600  0.31493600  -0.67350200  0.82274900  0.95898500  2.01033400  -3.16100900  -2.08088800  -0.41467700  -1.91154100  -2.66357600  -1.26584800  -1.17968200  0.33541200  1.59096500  1.99890000  1.43605300  2.50627100  -0.89491800  0.59921800  0.99140900  1.19488800  0.98343100  0.60423800  -0.78471200  -1.83782900  -1.46661900  -0.08350800  -1.04888800  0.61333000  1.35478200  1.05616900  1.95724600  -1.91981800  -2.81983600  -2.21044500  -1.45768600  -0.08483300  -0.76735300  0.31721400  -0.25110300  1.37714300  1.61474300 |
| (C) [C4COOHMIM][BF_4_]-CHCA | | | | (D) [C4COOCMIM][BF_4_]-CHCA | | | |
| N  C  C  N  C  C  C  C  C  C  H  H  H  H  H  H  H  H  H  H  H  H  H  H  C  O  O  H  F  B  F  F  F  C  C  C  C  C  C  H  H  H  H  H  H  H  H  H  H  H  C  O  O  H | -2.16857200  -3.47565300  -3.48946200  -2.19255200  -1.41651700  -1.75092200  -1.70894100  -2.35876700  -1.35766800  -0.94781300  -4.27295300  -4.30036300  -0.35932000  -0.68341800  -1.95921300  -2.28443100  -1.94373200  -0.62854900  -2.95417700  -3.04742500  -1.81902900  -0.46754100  -0.43582000  -1.82162000  -0.03261900  0.39933800  0.29349800  0.81034500  -3.43795400  -2.40045700  -1.15947700  -2.47336300  -2.38915700  4.32943500  5.63849300  5.86249000  4.67784600  3.36624400  3.14490200  6.78817300  5.60410800  6.47420200  4.38519900  4.15844200  4.61009700  4.83543800  2.51697000  3.39084200  3.04632500  5.98373400  1.87266600  1.32983200  1.40381200  0.50930400 | 0.74979100  0.35168900  -1.00230900  -1.40641000  -0.33028300  -2.79137500  2.13802300  2.87606200  3.56361100  2.72615800  1.06114300  -1.70747900  -0.33978900  -2.77931700  -3.33931800  -3.21655500  2.62599100  2.09251000  2.16143400  3.62625300  4.47601100  3.87885300  3.34496800  2.30090400  1.56955500  1.27522200  0.91365000  0.12248100  -1.89790400  -1.18583100  -1.79744000  -1.20411200  0.14027000  -1.26177600  -0.47055800  0.26929800  1.18122600  0.39527700  -0.33349500  0.84898900  0.25584500  -1.14388300  -2.04373800  -1.75506300  1.97090600  1.67851000  1.05599700  -0.34479400  0.39953900  -0.46451700  -1.13687900  -1.54782200  -1.39999400  -1.80151400 | -1.80699200  -1.98557000  -1.91498000  -1.70041500  -1.61538500  -1.53415800  -1.70167200  -0.51344800  0.43299900  1.64190100  -2.12327200  -1.96232700  -1.39478400  -1.32724800  -2.45281000  -0.68678400  -2.64937200  -1.58900500  0.05917500  -0.90826600  0.81724700  -0.12060600  2.38498900  2.14349100  1.34573400  0.24973400  2.45106200  2.20988700  0.86630800  1.47289900  1.04500800  2.84170500  0.97957000  0.43664400  0.45801100  -0.86259900  -1.18988600  -1.21861400  0.10558200  -0.81822000  1.27880600  0.66508900  -0.33022300  1.39646800  -0.43159500  -2.15038100  -1.41030600  -2.02538700  0.91481200  -1.66858600  0.08067100  -0.91722100  1.31001200  1.23314600 | N  C  C  N  C  C  C  C  C  C  H  H  H  H  H  H  H  H  H  H  H  H  H  H  C  O  O  C  H  H  H  F  B  F  F  F  C  C  C  C  C  C  H  H  H  H  H  H  H  H  H  H  H  C  O  O  H | -2.35144000  -3.69633300  -4.29653600  -3.30721100  -2.14517100  -3.51783200  -1.30443400  -1.75467000  -0.60338300  -0.12622400  -4.10914100  -5.33026400  -1.18245200  -2.56164600  -4.22125000  -3.89299500  -1.06917800  -0.42697300  -2.11610300  -2.59480600  -0.92421100  0.24424400  0.61719500  -0.94889600  0.53889400  1.12902500  0.44037300  1.00542100  2.04786000  0.92530900  0.43368900  -1.49411100  -2.04794800  -3.38053100  -1.30911500  -1.91124000  3.92365700  5.38183800  5.66380500  4.70783200  3.24862900  2.97385500  6.70229800  5.59277900  6.04713100  3.72618900  3.72144200  4.89488600  4.89891900  2.56859500  3.02860700  3.12208500  5.53566700  1.54316500  0.78078800  1.20758900  0.25902300 | -0.10263500  0.01242700  -1.10676100  -1.88743000  -1.25281300  -3.07631900  0.90796700  2.27763100  3.30329600  3.71446400  0.86978000  -1.40512700  -1.58116700  -3.57553000  -3.73728000  -2.75815600  0.92918300  0.56933500  2.15771100  2.63094300  4.19985200  2.89182800  4.51551200  4.09573800  2.57838400  1.66947900  2.73917800  1.70335200  1.53591000  2.06207700  0.78857600  -1.73479000  -0.41542300  -0.44589700  0.44932300  -0.05017400  -1.88836800  -1.45718500  -0.98921400  0.13395300  -0.29549900  -0.75009600  -0.65965000  -0.63630800  -2.28156000  -2.76237100  -2.18064200  1.01339700  0.43761800  0.52060200  -1.12519600  0.09195800  -1.83578500  -1.18815300  -1.42248800  -1.34959200  -1.58483600 | -1.77251700  -2.05284100  -1.57418100  -1.02060900  -1.14100500  -0.19973600  -1.99526000  -1.49845100  -1.54916000  -0.15933900  -2.55372300  -1.56405900  -0.76902200  -0.06490700  -0.70353200  0.77208700  -3.06235400  -1.44275100  -0.47381300  -2.10262700  -2.08248500  -2.10526600  -0.22925300  0.44810500  0.58693900  0.05451000  1.90020400  2.71470100  2.44193000  3.73707700  2.58348500  1.59425000  1.78960400  1.37099900  0.95034200  3.10766100  0.41962800  0.25176000  -1.17764400  -1.58629400  -1.42782900  0.00339200  -1.26974900  0.94792400  0.52212000  -0.21371800  1.45230600  -0.95776300  -2.61902700  -1.68028900  -2.10879400  0.68824000  -1.86336200  0.17969900  -0.73542000  1.45408600  1.51449600 |
| (E) [C4OCMIM][BF_4_]-CHCA | | | |  | | | |
| N  C  C  N  C  C  C  C  C  C  H  H  H  H  H  H  H  H  H  H  H  H  F  B  F  F  F  H  H  O  C  H  H  H  C  C  C  C  C  C  H  H  H  H  H  H  H  H  H  H  H  C  O  O  H | 1.68167100  2.57974300  1.93992700  0.66364900  0.53779900  -0.34298500  1.97526400  3.17868000  3.25795800  4.49017600  3.58773500  2.28089100  -0.35088600  -1.28080700  -0.44772800  -0.03214100  2.13178200  1.08311900  3.07182400  4.10281500  3.28032100  2.36938300  0.72334600  0.97604900  -0.18948200  2.05739200  1.17744800  4.49416200  4.49795900  5.63507400  6.82931900  6.91254500  7.65671400  6.88806200  -4.90418500  -6.16038700  -5.84005300  -5.16221800  -3.90548700  -4.23658600  -6.75087400  -6.90106300  -6.61407500  -4.18811200  -5.14353700  -5.86352000  -4.90555400  -3.44859300  -3.16538600  -4.90996300  -5.16944700  -2.96830700  -2.37191200  -2.55429400  -1.68433200 | 1.05358500  2.08524700  3.22662800  2.87010200  1.55271000  3.74121700  -0.38173100  -0.76105700  -2.27473700  -2.68390700  1.91773600  4.24644000  1.00020900  3.19031100  4.63806700  3.98458900  -0.62381500  -0.89655700  -0.27969500  -0.40140600  -2.77547000  -2.62201000  -0.27741200  0.85420200  1.70031500  1.56708400  0.49900000  -2.18992700  -3.76997600  -2.30428400  -2.61685300  -3.69660400  -2.29547400  -2.09623400  -0.11441600  -0.97310500  -2.45948300  -2.72120200  -1.86612200  -0.37101800  -3.05744600  -0.67459800  -0.79353800  -0.35174500  0.94711900  -2.48659100  -3.77901000  -2.02695300  -2.15190800  -0.07891300  -2.77821700  0.42972500  0.96015400  0.46569100  0.92004600 | -1.51639300  -1.67884500  -1.31943200  -0.94616800  -1.06094200  -0.34410400  -1.62673000  -0.77246800  -0.59862900  0.18452100  -2.01513700  -1.28199400  -0.79026000  -0.30846400  -0.95287900  0.67061300  -2.68065500  -1.27035300  0.20210100  -1.23281500  -1.57280300  -0.06235600  1.04664100  1.85733300  1.74756900  1.32470000  3.16534400  1.16721100  0.35187700  -0.54951400  0.12377000  0.30198000  -0.50746200  1.08771400  -1.02882000  -1.18616400  -1.01279800  0.33379700  0.49943500  0.32092100  -1.10119000  -0.43413800  -2.16422600  -1.82265100  -1.13183100  1.14356400  0.43338800  1.47780600  -0.25851900  1.13568400  -1.82011100  0.48761900  -0.42933700  1.74209500  1.79616700 |  |  |  |  |

Supplementary Table S3. The NBO charge of (A) [C4OHMIM][BF_4_], (B) [C4NHMIM][BF_4_], (C) [C4COOHMIM][BF_4_], (D) [C4COOCMIM][BF_4_], (E) [C4OCMIM][BF_4_], (F) [C4OHMIM][BF4]-CHCA, (G) [C4NHMIM][BF_4_]-CHCA, (H) [C4COOHMIM][BF_4_]-CHCA, (I) [C4COOCMIM][BF_4_]-CHCA, and (J) [C4OCMIM][BF_4_]-CHCA.

| A | | B | | C | | | D | | E | |
| --- | --- | --- | --- | --- | --- | --- | --- | --- | --- | --- |
| N1 | -0.36618 | N1 | -0.36619 | N1 | | -0.36939 | N1 | -0.36814 | N1 | -0.36613 |
| C2 | -0.01966 | C2 | -0.01957 | C2 | | -0.02524 | C2 | -0.02423 | C2 | -0.01971 |
| C3 | -0.01712 | C3 | -0.01607 | C3 | | -0.00032 | C3 | -0.00204 | C3 | -0.01696 |
| N4 | -0.36617 | N4 | -0.36591 | N4 | | -0.36389 | N4 | -0.36476 | N4 | -0.36614 |
| C5 | 0.33827 | C5 | 0.33649 | C5 | | 0.33543 | C5 | 0.33689 | C5 | 0.33834 |
| C6 | -0.35163 | C6 | -0.35259 | C6 | | -0.36221 | C6 | -0.36021 | C6 | -0.35160 |
| C7 | -0.16071 | C7 | -0.16030 | C7 | | -0.17057 | C7 | -0.17487 | C7 | -0.16048 |
| C8 | -0.42475 | C8 | -0.43210 | C8 | | -0.42669 | C8 | -0.42102 | C8 | -0.42334 |
| C9 | -0.41055 | C9 | -0.39859 | C9 | | -0.38720 | C9 | -0.38602 | C9 | -0.40831 |
| C10 | -0.02038 | C10 | -0.17614 | C10 | | -0.49482 | C10 | -0.49692 | C10 | -0.01317 |
| H11 | 0.23856 | H11 | 0.23751 | H11 | | 0.23423 | H11 | 0.23382 | H11 | 0.23842 |
| H12 | 0.23383 | H12 | 0.23402 | H12 | | 0.24028 | H12 | 0.23929 | H12 | 0.23383 |
| H13 | 0.27261 | H13 | 0.27133 | H13 | | 0.27291 | H13 | 0.27246 | H13 | 0.27260 |
| H14 | 0.22873 | H14 | 0.22805 | H14 | | 0.23254 | H14 | 0.22603 | H14 | 0.22857 |
| H15 | 0.20170 | H15 | 0.20208 | H15 | | 0.19981 | H15 | 0.19915 | H15 | 0.20169 |
| H16 | 0.24386 | H16 | 0.24537 | H16 | | 0.25270 | H16 | 0.25691 | H16 | 0.24400 |
| H17 | 0.20408 | H17 | 0.20360 | H17 | | 0.20422 | H17 | 0.20371 | H17 | 0.20396 |
| H18 | 0.23303 | H18 | 0.22833 | H18 | | 0.24325 | H18 | 0.24682 | H18 | 0.23334 |
| H19 | 0.25036 | H19 | 0.26956 | H19 | | 0.24643 | H19 | 0.24572 | H19 | 0.25073 |
| H20 | 0.20987 | H20 | 0.19353 | H20 | | 0.20490 | H20 | 0.20291 | H20 | 0.20846 |
| H21 | 0.19725 | H21 | 0.19488 | H21 | | 0.20985 | H21 | 0.21047 | H21 | 0.19771 |
| H22 | 0.22228 | H22 | 0.21398 | H22 | | 0.20488 | H22 | 0.20241 | H22 | 0.22219 |
| F23 | -0.59525 | H23 | 0.17359 | H23 | | 0.23472 | H23 | 0.23132 | F23 | -0.59526 |
| B24 | 1.34972 | F24 | -0.59883 | H24 | | 0.24881 | H24 | 0.26938 | B24 | 1.34975 |
| F25 | -0.58979 | B25 | 1.34946 | C25 | | 0.86864 | C25 | 0.87465 | F25 | -0.58991 |
| F26 | -0.58936 | F26 | -0.58635 | O26 | | -0.68976 | O26 | -0.68947 | F26 | -0.58936 |
| F27 | -0.54646 | F27 | -0.58400 | O27 | | -0.69256 | O27 | -0.54920 | F27 | -0.54632 |
| H28 | 0.16043 | F28 | -0.54981 | H28 | | 0.51843 | C28 | -0.21706 | H28 | 0.16456 |
| H29 | 0.16545 | H29 | 0.16391 | F29 | | -0.57595 | H29 | 0.17449 | H29 | 0.16848 |
| O30 | -0.75493 | N30 | -0.87253 | B30 | | 1.35752 | H30 | 0.18917 | O30 | -0.61501 |
| H31 | 0.46291 | H31 | 0.37678 | F31 | | -0.60678 | H31 | 0.21076 | C31 | -0.19682 |
|  |  | H32 | 0.35651 | F32 | | -0.54798 | F32 | -0.60000 | H32 | 0.15763 |
|  |  |  |  | F33 | | -0.59618 | B33 | 1.35662 | H33 | 0.18335 |
|  |  |  |  |  | |  | F34 | -0.57777 | H34 | 0.16090 |
|  |  |  |  |  | |  | F35 | -0.60047 |  |  |
|  |  |  |  |  | |  | F36 | -0.55079 |  |  |
| F | | G | | H | | | I | | J | |
| N1 | -0.36773 | N1 | -0.36797 | N1 | -0.36985 | | N1 | -0.36765 | N1 | -0.36771 |
| C2 | -0.01865 | C2 | -0.01834 | C2 | -0.03167 | | C2 | -0.02799 | C2 | -0.01822 |
| C3 | -0.01794 | C3 | -0.01680 | C3 | 0.00247 | | C3 | -0.01725 | C3 | -0.01786 |
| N4 | -0.36678 | N4 | -0.36727 | N4 | -0.36327 | | N4 | -0.36765 | N4 | -0.36679 |
| C5 | 0.32694 | C5 | 0.32549 | C5 | 0.32613 | | C5 | 0.32286 | C5 | 0.32644 |
| C6 | -0.36075 | C6 | -0.36169 | C6 | -0.36785 | | C6 | -0.35555 | C6 | -0.36035 |
| C7 | -0.15982 | C7 | -0.15973 | C7 | -0.17381 | | C7 | -0.17815 | C7 | -0.15944 |
| C8 | -0.42409 | C8 | -0.43214 | C8 | -0.42354 | | C8 | -0.41656 | C8 | -0.42254 |
| C9 | -0.40904 | C9 | -0.39805 | C9 | -0.38726 | | C9 | -0.39117 | C9 | -0.40668 |
| C10 | -0.02024 | C10 | -0.17676 | C10 | -0.49584 | | C10 | -0.47706 | C10 | -0.01302 |
| H11 | 0.23688 | H11 | 0.23612 | H11 | 0.23316 | | H11 | 0.23345 | H11 | 0.23669 |
| H12 | 0.23231 | H12 | 0.23252 | H12 | 0.23977 | | H12 | 0.23235 | H12 | 0.23243 |
| H13 | 0.28877 | H13 | 0.28788 | H13 | 0.27967 | | H13 | 0.29666 | H13 | 0.28892 |
| H14 | 0.24554 | H14 | 0.24458 | H14 | 0.24807 | | H14 | 0.22810 | H14 | 0.24516 |
| H15 | 0.20088 | H15 | 0.20115 | H15 | 0.19832 | | H15 | 0.20216 | H15 | 0.20102 |
| H16 | 0.23358 | H16 | 0.23498 | H16 | 0.24555 | | H16 | 0.24317 | H16 | 0.23343 |
| H17 | 0.20328 | H17 | 0.20316 | H17 | 0.20388 | | H17 | 0.20095 | H17 | 0.20319 |
| H18 | 0.23161 | H18 | 0.22697 | H18 | 0.24633 | | H18 | 0.27129 | H18 | 0.23135 |
| H19 | 0.24605 | H19 | 0.26581 | H19 | 0.24384 | | H19 | 0.23804 | H19 | 0.24658 |
| H20 | 0.21053 | H20 | 0.19420 | H20 | 0.20358 | | H20 | 0.19449 | H20 | 0.20917 |
| H21 | 0.19844 | H21 | 0.19488 | H21 | 0.21048 | | H21 | 0.20777 | H21 | 0.19888 |
| H22 | 0.21790 | H22 | 0.21364 | H22 | 0.20404 | | H22 | 0.21312 | H22 | 0.21735 |
| F23 | -0.58755 | H23 | 0.17429 | H23 | 0.23328 | | H23 | 0.23170 | F23 | -0.58761 |
| B24 | 1.35394 | F24 | -0.59202 | H24 | 0.25656 | | H24 | 0.22815 | B24 | 1.35382 |
| F25 | -0.59611 | B25 | 1.35519 | C25 | 0.86910 | | C25 | 0.86345 | F25 | -0.59596 |
| F26 | -0.57575 | F26 | -0.59165 | O26 | -0.69364 | | O26 | -0.65796 | F26 | -0.57554 |
| F27 | -0.54467 | F27 | -0.57132 | O27 | -0.70004 | | O27 | -0.55834 | F27 | -0.54449 |
| F28 | 0.16410 | F28 | -0.54913 | H28 | 0.51321 | | C28 | -0.21354 | H28 | 0.16722 |
| H29 | 0.16009 | H29 | 0.16362 | F29 | -0.57094 | | H29 | 0.17424 | H29 | 0.16414 |
| O30 | -0.75420 | N30 | -0.87252 | B30 | 1.36634 | | H30 | 0.18712 | O30 | -0.61427 |
| H31 | 0.46306 | H31 | 0.37475 | F31 | -0.61088 | | H31 | 0.21504 | C31 | -0.19686 |
| C32 | -0.39055 | H32 | 0.35636 | F32 | -0.54588 | | F32 | -0.59752 | H32 | 0.15751 |
| C33 | -0.39001 | C33 | -0.38236 | F33 | -0.59280 | | B33 | 1.35477 | H33 | 0.18386 |
| C34 | -0.39190 | C34 | -0.39062 | C34 | -0.38105 | | F34 | -0.57331 | H34 | 0.16078 |
| C35 | -0.39050 | C35 | -0.39191 | C35 | -0.38966 | | F35 | -0.59022 | C35 | -0.39057 |
| C36 | -0.38206 | C36 | -0.39020 | C36 | -0.39173 | | F36 | -0.54804 | C36 | -0.39010 |
| C37 | -0.32010 | C37 | -0.39039 | C37 | -0.38910 | | C37 | -0.38142 | C37 | -0.39174 |
| H38 | 0.20430 | C38 | -0.32037 | C38 | -0.39153 | | C38 | -0.38894 | C38 | -0.39048 |
| H39 | 0.19160 | H39 | 0.20455 | C39 | -0.32227 | | C39 | -0.39109 | C39 | -0.38202 |
| H40 | 0.20506 | H40 | 0.19413 | H40 | 0.20450 | | C40 | -0.38852 | C40 | -0.32013 |
| H41 | 0.20803 | H41 | 0.20672 | H41 | 0.19391 | | C41 | -0.39293 | H41 | 0.20420 |
| H42 | 0.21081 | H42 | 0.19609 | H42 | 0.20732 | | C42 | -0.32317 | H42 | 0.19169 |
| H43 | 0.19375 | H43 | 0.22170 | H43 | 0.20065 | | H43 | 0.20192 | H43 | 0.20501 |
| H44 | 0.20697 | H44 | 0.19198 | H44 | 0.21648 | | H44 | 0.19240 | H44 | 0.20789 |
| H45 | 0.22313 | H45 | 0.20519 | H45 | 0.19208 | | H45 | 0.20418 | H45 | 0.21087 |
| H46 | 0.19699 | H46 | 0.21146 | H46 | 0.20501 | | H46 | 0.19571 | H46 | 0.19379 |
| H47 | 0.22468 | H47 | 0.20747 | H47 | 0.21939 | | H47 | 0.21785 | H47 | 0.20690 |
| H48 | 0.19184 | H48 | 0.22599 | H48 | 0.20787 | | H48 | 0.19173 | H48 | 0.22362 |
| C49 | 0.86303 | H49 | 0.19192 | H49 | 0.22096 | | H49 | 0.20210 | H49 | 0.19684 |
| O50 | -0.68785 | C50 | 0.86380 | H50 | 0.19278 | | H50 | 0.22626 | H50 | 0.22470 |
| O51 | -0.70700 | O51 | -0.68769 | C51 | 0.88428 | | H51 | 0.20217 | H51 | 0.19167 |
| H52 | 0.52920 | O52 | -0.70729 | O52 | -0.66031 | | H52 | 0.23184 | C52 | 0.86344 |
|  |  | H53 | 0.52962 | O53 | -0.75502 | | H53 | 0.19089 | O53 | -0.68803 |
|  |  |  |  | H54 | 0.53893 | | C54 | 0.88817 | O54 | -0.70687 |
|  |  |  |  |  |  | | O55 | -0.68602 | H 55 | 0.52873 |
|  |  |  |  |  |  | | O56 | -0.71675 |  |  |
|  |  |  |  |  |  | | H57 | 0.52272 |  |  |

Supplementary Table S4. The donor-acceptor interaction in [C4OHMIM][BF_4_]-CHCA, [C4NHMIM][BF_4_]-CHCA, [C4COOHMIM][BF_4_]-CHCA, [C4COOCMIM][BF_4_]-CHCA, [C4OCMIM][BF_4_]-CHCA, and their second order perturbation stabilization energies, *E*(2)(kcal/mol).

| Donor | Acceptor | *E* (kcal/mol) | Donor | Acceptor | *E* (kcal/mol) |
| --- | --- | --- | --- | --- | --- |
| [C4OHMIM][BF_4_]-CHCA | | | | | |
| σ(C5-H13) | σ*(C49-O50) | 0.11 | LP(O50) | π*(N1-C5) | 0.25 |
| σ(C6-H14) | σ*(C49-O50) | 0.07 | LP(O50) | σ*(C5-H13) | 5.45 |
| σ(B24-F25) | σ*(O51-H52) | 0.11 | LP(O50) | σ*(C6-H14) | 0.83 |
| LP(F25) | σ*(O51-H52) | 20.18 | σ*(C49-O50) | σ*(C5-H13) | 0.14 |
| σ(C37–C49) | σ*(C5-H13) | 0.25 | σ*(C49-O50) | σ*(C6-H14) | 0.07 |
| σ(C49-O50) | σ*(C5-H13) | 1.03 | σ(O51-H52) | σ*(B24-F25) | 0.05 |
| σ(C49-O50) | σ*(C6-H14) | 0.36 | LP(O51) | σ*(B24-F25) | 0.09 |
| [C4NHMIM][BF_4_]-CHCA | | | | | |
| σ(C5-H13) | σ*(C50-O51) | 0.12 | LP(O51) | σ*(C5-H13) | 1.97 |
| σ (C6-H14) | σ*(C49-O50) | 0.07 | LP(O51) | σ*(C6-H14) | 0.78 |
| σ(B25-F26) | σ*(O52-H53) | 0.10 | LP(O51) | π*(N1-C5) | 0.13 |
| LP(F26) | σ*(O52-H53) | 20.31 | σ*(C50-O51) | σ*(C5-H13) | 0.23 |
| σ(C38–C50) | σ*(C5-H13) | 0.25 | σ*(C50-O51) | σ*(C6-H14) | 0.05 |
| σ(C50-O51) | σ*(C5-H13) | 0.98 | σ (O52-H52) | σ*(B25-F26) | 0.06 |
| σ(C50-O51) | σ*(C6-H14) | 0.35 | LP(O52) | σ*(B25-F26) | 0.09 |
| [C4COOHMIM][BF_4_]-CHCA | | | | | |
| σ(C5-H13) | σ*(C51-O52) | 0.23 | σ(C51-O52) | σ*(O27-H28) | 0.16 |
| σ(O27-H28) | σ*(C51-O53) | 0.06 | σ(C51-O52) | π*(N1-C5) | 0.08 |
| LP(O26) | σ*(C34-C39) | 0.20 | σ(C51-O53) | σ*(C5-H13) | 0.09 |
| LP(O26) | σ*(C38-H47) | 0.15 | σ(O53-H54) | σ*(O27-H28) | 0.29 |
| LP(O26) | σ*(C51-O52) | 2.44 | LP(O52) | π*(N1-C5) | 0.07 |
| LP(O26) | σ*(C51-O53) | 0.14 | LP(O52) | σ*(C5-H13) | 2.07 |
| LP(F31) | σ*(O53-H54) | 18.09 | LP(O52) | σ*(C6-H14) | 1.65 |
| σ(C38-H47) | σ*(C5-H13) | 0.07 | LP(O53) | σ*(O27-H28) | 11.56 |
| σ(C39-C51) | σ*(C5-H13) | 0.18 | σ*(C51-O52) | σ*(C5-H13) | 0.27 |
| σ(C39-C51) | σ*(C6-H14) | 0.06 | σ(O53-H54) | σ*(B30-F31) | 0.15 |
| σ(C51-O52) | σ*(C5-H13) | 2.10 | LP(O53) | σ*(B30-F31) | 0.12 |
| [C4COOCMIM][BF_4_]-CHCA | | | | | |
| σ(C5-H13) | σ*(C54-O55) | 0.07 | σ(C42-C54) | σ*(C5-H13) | 0.39 |
| σ(C7-H18) | σ*(O54-O55) | 0.11 | σ(C54-O55) | σ*(C5-H13) | 0.25 |
| σ(C25-O26) | σ*(C54-O55) | 0.12 | σ(C54-O55) | π*(N4-C5) | 0.06 |
| σ(C28-H29) | σ*(C42-H52) | 0.25 | σ(C54-O55) | σ*(C7-H18) | 0.82 |
| LP(O26) | σ*(C37-C42) | 0.37 | σ(C54-O55) | σ*(C25-O56) | 0.08 |
| LP(O26) | σ*(C41-H50) | 0.36 | σ(C56-H57) | σ*(C28-H31) | 0.06 |
| LP(O26) | σ*(C41-H51) | 0.06 | LP(O55) | π*(N4-C5) | 0.21 |
| LP(O26) | σ*(C42-H52) | 0.07 | LP(O55) | σ*(C5-H13) | 10.26 |
| LP(O26) | σ*(C54-O55) | 1.14 | LP(O55) | σ*(C7-H18) | 0.40 |
| σ(F32-B33) | σ*(O56-H57) | 0.15 | LP(O56) | σ*(C28-H31) | 0.55 |
| LP(F32) | σ*(O56-H57) | 14.99 | σ*(C54-O55) | σ*(C7-H18) | 0.08 |
| σ(C42-H52) | σ*(C28-H29) | 0.12 | LP(O56) | σ*(F32-B33) | 0.07 |
| [C4OCMIM][BF_4_]-CHCA | | | | | |
| σ(C5-H13) | σ*(C52-O53) | 0.13 | LP(O53) | π*(N1-C5) | 0.23 |
| σ(C6-H14) | σ*(C52-O53) | 0.05 | LP(O53) | σ*(C5-H13) | 5.61 |
| σ(B24-F25) | σ*(O54-H55) | 0.29 | LP(O53) | σ*(C6-H14) | 0.80 |
| LP(F25) | σ*(O54-H55) | 20.35 | σ*(C52-O53) | σ*(C5-H13) | 0.17 |
| σ(C40-C52) | σ*(C5-H13) | 0.26 | σ*(C52-O53) | σ*(C6-H14) | 0.06 |
| σ(C52-O53) | σ*(C5-H13) | 1.19 | σ(O54-H55) | σ*(B24-F25) | 0.05 |
| σ(C52-O53) | σ*(C6-H14) | 0.28 | LP(O54) | σ*(B24-F25) | 0.09 |

Supplementary Table S5. Electron densities (ρ) and Laplacians of electron density (∇^2^ρ) of BCPs in (A) [C4OHMIM][BF_4_], (B) [C4NHMIM][BF_4_], (C) [C4COOHMIM][BF_4_], (D) [C4COOCMIM][BF_4_], (E) [C4OCMIM][BF_4_], (F) [C4OHMIM][BF_4_]-CHCA, (G) [C4NHMIM][BF_4_]-CHCA, (H) [C4COOHMIM][BF_4_]-CHCA, (I) [C4COOCMIM][BF_4_]-CHCA, and, (J) [C4OCMIM][BF_4_]-CHCA.

| CP label | X···Y | ρ (a.u.) | ∇^2^ρ(a.u.) |
| --- | --- | --- | --- |
| (A) [C4OHMIM][BF_4_] | | | |
| 53 | F26···C5 | 0.1387E-01 | 0.5513E-01 |
| 56 | F23···H18 | 0.1209E-01 | 0.4838E-01 |
| 58 | F23···H13 | 0.1401E-01 | 0.5828E-01 |
| 59 | F25···H13 | 0.1498E-01 | 0.6191E-01 |
| 64 | F25···C6 | 0.1088E-01 | 0.4746E-01 |
| 66 | F26···H18 | 0.9306E-02 | 0.3376E-01 |
| 67 | F23···H19 | 0.1076E-01 | 0.4173E-01 |
| (B)[C4NHMIM][BF_4_] | | | |
| 54 | F27···H19 | 0.9309E-02 | 0.3415E-01 |
| 58 | F24···H19 | 0.1149E-01 | 0.4625E-01 |
| 61 | F24···H18 | 0.1122E-01 | 0.4582E-01 |
| 65 | F27···C5 | 0.1441E-01 | 0.5706E-01 |
| 66 | F24···H22 | 0.1066E-01 | 0.4160E-01 |
| 71 | F24···H13 | 0.1420E-01 | 0.5899E-01 |
| 75 | F24···H31 | 0.6897E-02 | 0.2748E-01 |
| 78 | F26···H13 | 0.1348E-01 | 0.5550E-01 |
| 83 | F28···H31 | 0.5676E-02 | 0.2362E-01 |
| 85 | F26···C6 | 0.1034E-01 | 0.4442E-01 |
| (C) [C4COOHMIM][BF_4_] | | | |
| 49 | O26···H18 | 0.1534E-01 | 0.5598E-01 |
| 54 | O26···H13 | 0.1710E-01 | 0.7209E-01 |
| 60 | F33···N1 | 0.1208E-01 | 0.4952E-01 |
| 61 | F29···N4 | 0.1094E-01 | 0.4570E-01 |
| 63 | F33···N4 | 0.1018E-01 | 0.4177E-01 |
| 66 | F33···H19 | 0.1171E-01 | 0.4686E-01 |
| 69 | F31···H14 | 0.1118E-01 | 0.4730E-01 |
| 71 | F29···H16 | 0.1482E-01 | 0.5883E-01 |
| 76 | F33···C25 | 0.1151E-01 | 0.4832E-01 |
| 85 | F31···H28 | 0.2072E-01 | 0.8397E-01 |
| (D) [C4COOCMIM][BF_4_] | | | |
| 48 | O26···H18 | 0.1559E-01 | 0.5611E-01 |
| 61 | O26···H13 | 0.1576E-01 | 0.6438E-01 |
| 62 | F35···H19 | 0.1255E-01 | 0.5107E-01 |
| 63 | F35···N1 | 0.1303E-01 | 0.5448E-01 |
| 75 | F32···C5 | 0.1212E-01 | 0.4962E-01 |
| 76 | F35···H24 | 0.1271E-01 | 0.4885E-01 |
| 78 | F34···N4 | 0.9173E-02 | 0.3944E-01 |
| 84 | F32···C6 | 0.1167E-01 | 0.5220E-01 |
| 87 | F34···H16 | 0.1459E-01 | 0.5671E-01 |
| 91 | F32···H31 | 0.1427E-01 | 0.5684E-01 |
| (E) [C4OCMIM][BF_4_] | | | |
| 56 | F26···C5 | 0.1381E-01 | 0.5488E-01 |
| 58 | F23···H18 | 0.1211E-01 | 0.4842E-01 |
| 61 | F23···H13 | 0.1401E-01 | 0.5829E-01 |
| 62 | F25···H13 | 0.1497E-01 | 0.6191E-01 |
| 67 | F25···C6 | 0.1093E-01 | 0.4762E-01 |
| 71 | F26···H19 | 0.9325E-02 | 0.3382E-01 |
| 72 | F23···H19 | 0.1063E-01 | 0.4128E-01 |
| (F) [C4OHMIM][BF_4_]-CHCA | | | |
| 58 | O50···H13 | 0.9534E-02 | 0.4119E-01 |
| 62 | O50···H14 | 0.9571E-02 | 0.3142E-01 |
| 66 | F25···C5 | 0.1003E-01 | 0.4191E-01 |
| 68 | F26···C5 | 0.1181E-01 | 0.4856E-01 |
| 71 | F25···H52 | 0.3653E-01 | 0.1526E+00 |
| 82 | F25···H16 | 0.1903E-01 | 0.7802E-01 |
| 83 | F26···H19 | 0.9821E-02 | 0.3560E-01 |
| 84 | F23···C5 | 0.1248E-01 | 0.5121E-01 |
| 92 | F23···H19 | 0.9386E-02 | 0.3661E-01 |
| 97 | F23···H18 | 0.1116E-01 | 0.4501E-01 |
| (G) [C4NHMIM][BF_4_]-CHCA | | | |
| 59 | F28···H31 | 0.7535E-02 | 0.2965E-01 |
| 65 | F24···H31 | 0.4673E-02 | 0.2078E-01 |
| 72 | F24···H22 | 0.1019E-01 | 0.3909E-01 |
| 82 | F26···H35 | 0.3647E-01 | 0.1528E+00 |
| 92 | F24···H19 | 0.1013E-01 | 0.4128E-01 |
| 97 | F24···H18 | 0.1042E-01 | 0.4337E-01 |
| 101 | F27···H19 | 0.1048E-01 | 0.3847E-01 |
| 103 | F24··C5 | 0.1316E-01 | 0.5365E-01 |
| 111 | F26···C5 | 0.7603E-02 | 0.3396E-01 |
| 113 | O51···H13 | 0.1914E-01 | 0.7901E-01 |
| 114 | F27···C5 | 0.1227E-01 | 0.5022E-01 |
| 115 | F26···H16 | 0.8502E-02 | 0.3557E-01 |
| 120 | O51···H14 | 0.9461E-02 | 0.3129E-01 |
| (H) [C4COOHMIM][BF_4_]-CHCA | | | |
| 56 | F29···H16 | 0.1152E-01 | 0.4446E-01 |
| 59 | F31···C6 | 0.1151E-01 | 0.5138E-01 |
| 64 | F31···H54 | 0.3689E-01 | 0.1606E+00 |
| 68 | O52···H14 | 0.1049E-01 | 0.3529E-01 |
| 70 | F29···N4 | 0.1033E-01 | 0.4182E-01 |
| 82 | O53···H28 | 0.2841E-01 | 0.1097E+00 |
| 85 | O52···H13 | 0.1609E-01 | 0.6472E-01 |
| 95 | F33···C5 | 0.1203E-01 | 0.4882E-01 |
| 98 | O26···C51 | 0.1134E-01 | 0.4283E-01 |
| 103 | F25···C25 | 0.1038E-01 | 0.4432E-01 |
| 104 | O26···H13 | 0.1041E-01 | 0.3934E-01 |
| 110 | F33···H24 | 0.9388E-02 | 0.3893E-01 |
| 115 | F33···H19 | 0.1152E-01 | 0.4591E-01 |
| 122 | O26···H47 | 0.6241E-02 | 0.2231E-01 |
| 130 | O26···H18 | 0.1594E-01 | 0.5824E-01 |
| (I) [C4COOCMIM][BF_4_]-CHCA | | | |
| 61 | F32···C6 | 0.7957E-02 | 0.3508E-01 |
| 64 | F34···H16 | 0.9748E-02 | 0.3976E-01 |
| 67 | F34···H16 | 0.3089E-01 | 0.1306E+00 |
| 71 | F32···H13 | 0.1266E-01 | 0.5116E-01 |
| 74 | F34···H16 | 0.1239E-01 | 0.5000E-01 |
| 79 | O55···H13 | 0.2361E-01 | 0.9804E-01 |
| 94 | F35···O55 | 0.4746E-02 | 0.1969E-01 |
| 95 | F35···C5 | 0.1147E-01 | 0.4610E-01 |
| 97 | O56···H31 | 0.8688E-02 | 0.2873E-01 |
| 101 | F36···H31 | 0.7013E-02 | 0.2711E-01 |
| 108 | O55···H18 | 0.9560E-02 | 0.3203E-01 |
| 111 | F35···H31 | 0.9752E-02 | 0.3707E-01 |
| 113 | O26···C54 | 0.8920E-02 | 0.3741E-01 |
| 117 | F35···H18 | 0.9016E-02 | 0.3910E-01 |
| 119 | O26···H52 | 0.9163E-02 | 0.3550E-01 |
| 127 | F35···O26 | 0.9903E-02 | 0.4312E-01 |
| 130 | F35···H19 | 0.1101E-01 | 0.4219E-01 |
| 131 | O26···H18 | 0.1026E-01 | 0.3443E-01 |
| 132 | O26···H50 | 0.8367E-02 | 0.3068E-01 |
| (J) [C4OCMIM][BF_4_]-CHCA | | | |
| 85 | F23···H18 | 0.1097E-01 | 0.4431E-01 |
| 86 | F23···H19 | 0.9410E-02 | 0.3665E-01 |
| 101 | F25···H55 | 0.3678E-01 | 0.1534E+00 |
| 103 | F26···H19 | 0.9870E-02 | 0.3582E-01 |
| 104 | F23···C5 | 0.1261E-01 | 0.5168E-01 |
| 109 | O53···H13 | 0.1936E-01 | 0.7974E-01 |
| 114 | F25···C5 | 0.1001E-01 | 0.4173E-01 |
| 115 | F26···C5 | 0.1171E-01 | 0.4836E-01 |
| 118 | O53···H14 | 0.8928E-02 | 0.2894E-01 |
| 122 | F25···H16 | 0.9718E-02 | 0.4225E-01 |
